# Neural similarity and interaction success in autistic and non-autistic adolescents

**DOI:** 10.1038/s41598-025-91176-9

**Published:** 2025-03-07

**Authors:** Kathryn A. McNaughton, Sarah Dziura, Edward P. Lemay, Heather A. Yarger, Elizabeth Redcay

**Affiliations:** 1 Neuroscience and Cognitive Science Program, University of Maryland, 1121 Biology-Psychology Building, College Park, MD 20742 USA; 2 Department of Psychology, University of Maryland, 1121 Biology-Psychology Building, College Park, MD 20742 USA

**Keywords:** Social interaction, Autism, Adolescence, Neural similarity, fMRI, EMA, Social neuroscience, Autism spectrum disorders, Human behaviour

## Abstract

High-quality social interactions promote well-being for typically developing and autistic youth. One factor that may contribute to the quality of social interactions is neural similarity, a metric which may capture shared perspectives and experiences of the world. The current research investigates relations between neural similarity to peers and day-to-day interaction success as measured through ecological momentary assessment in a sample of autistic and non-autistic youth aged 11–14 years old. Neural similarity was operationalized as the between-participant correlation of participants’ neural response to naturalistic video stimuli in areas of the brain implicated in mental state understanding and reward processing. Neural similarity did not have a main effect on interaction success. However, across the full sample, neural similarity significantly interacted with reported closeness, such that there were more positive relations between neural similarity and interaction success for closer interactions. Neural similarity also marginally interacted with social partner (i.e., interactions featuring peers versus others) to predict interaction success, suggesting more positive relations between neural similarity and interaction success in peer interactions. In addition, non-autistic youth reported significantly better peer interactions than autistic youth. These findings suggest that similarity to one’s peers in neural processing in mentalizing and reward regions is important for understanding interaction success. They also highlight the challenge peer interactions may pose for autistic youth and propose novel links between peer interaction success and the brain’s mentalizing processes.

## Introduction

Navigating positive social interactions is a key component of mental health and well-being for youth^1^. Autistic youth often experience social rejection and challenges navigating social interactions^2^, which is associated with negative mental health outcomes and increased loneliness^3–5^. Past research has primarily used an individual-level approach to understanding how autism may impact social interactions. However, more recent scholarship has shifted focus to understanding interindividual processes, such as how similarity between individuals and their social worlds impact social interaction outcomes^6–8^. Links between dissimilarity and social interaction outcomes have not yet been examined at the neural level. A better understanding of how social interactions relate to neural dissimilarity has the potential to shed new light on understanding interpersonal challenges and successes for autistic and non-autistic youth.

Similarity is an interindividual factor that has a strong relation to people’s experiences in social interactions. People tend to feel connected and attracted to those who share their demographic and psychological characteristics, and these effects of similarity are especially pronounced with less familiar interaction partners^9–13^. Feeling a sense of similar understanding with others in the world, also known as generalized shared reality, likewise plays an important role in social connection through promoting rapport and interaction enjoyment^14,15^. Thus, the experience of similarity in understanding between oneself and one’s social world is a key shaper of social connection.

One means of quantifying similarity between people is neural similarity. Neural similarity provides an objective approach that more directly measures real-time similarity in cognitive and affective responses to stimuli than self-report instruments. Neural similarity is quantified by correlating the time series of each individual’s MRI BOLD response to stimuli between subjects using Pearson’s correlation^16,17^. Neural similarity indexes shared understanding and similar psychological perspectives^18^ such that participants who have more similar understanding of an event have higher neural similarity while viewing it^19–21^. In studies of neural similarity, participants view the stimuli at their own individual session. Hence, neural similarity represents the ‘offline’ similarity of individuals’ independent neural responses, rather than the neural response to an ‘online’ shared viewing experience.

Measures of neural similarity can be used to probe individuals’ shared understanding with others. Recent work has established links between neural similarity and experiences of one’s social world. That is, processing stimuli in similar ways to others (i.e. higher neural similarity to the group), particularly in brain regions associated with social cognition and thinking about others’ mental states (‘mentalizing’), is associated with higher popularity and reduced loneliness^22,23^. In contrast, idiosyncratic processing of stimuli is associated with reduced popularity and increased loneliness. In short, the relations between neural similarity and loneliness/popularity results follow an ‘Anna Karenina’ model^24^, named after Tolstoy’s famous line that ‘happy families are all alike, every unhappy family is unhappy in its own way’^25^. Specifically, individuals who are more popular and less lonely are more neurally similar to each other, while individuals who are less popular and more lonely are more neurally idiosyncratic^22,23^. This work provides a foundation for understanding relations between neural similarity and social experiences. However, no work has directly tested whether neural similarity relates to day-to-day experiences of social interactions. This includes relations to interaction success, how well an individual felt like their social interaction went, which is informative for understanding individuals’ perceptions of their social experiences with impacts on their broader well-being.

An individual’s autism diagnosis represents one potential dimension on which individuals’ similarity or dissimilarity may influence social interaction success. In laboratory-based ‘getting-to-know-you’ interactions, people enjoy the interaction more and want to interact with a partner again when paired with someone of the same neurotype (autistic with autistic, neurotypical with neurotypical) than when paired with someone of a different neurotype (autistic with neurotypical)^26–28^. In addition, autistic individuals differ from neurotypical peers in their neural response to stimuli. Autistic adults have reduced neural similarity with neurotypical adults and other autistic adults compared to levels of neural similarity observed among neurotypical adults^29–32^. Autistic children also have reduced neural similarity compared to neurotypical peers^33^. However, no work has linked these findings on neural dissimilarity in autistic individuals with research on dissimilarity in social interaction outcomes to directly test whether autistic individuals’ neural dissimilarity to others may predict reduced day-to-day interaction success and explain some of the social challenges associated with autism.

Early adolescence may be a particularly important time to investigate the role of neural similarity in social interaction outcomes, particularly social outcomes involving peers. During adolescence, friends become more similar to each other along several dimensions^34^, raising the possibility that similarity could be especially relevant for facilitating social connections with peers during this time. Additionally, early adolescence is an important time for understanding the successful navigation of social interactions. The transition to adolescence is a time of onset for many mental health conditions^35^, and positive peer encounters in this developmental period can promote mental resiliency^36^ and buffer against negative mental health outcomes^37^. Therefore, understanding factors like neural similarity that relate to social interaction outcomes in early adolescence may help support well-being in autistic and non-autistic youth during an important developmental period.

In the present study, we examine the role of neural similarity in interaction success in autistic and non-autistic early adolescents (11–14 years old). We tested the hypothesis that individuals who are more neurally similar to other individuals in the sample have better interaction success in day-to-day interactions measured through ecological momentary assessment (EMA). We further tested whether closeness to the social partner interacts with neural similarity in predicting interaction success. Given the findings noted previously suggesting that similarity is especially important when interacting with less familiar people, we predicted that the relation between interaction success and neural similarity would be stronger for less close interactions relative to closer interactions. Given the importance that peer interactions play in early adolescence, we also examined if neural similarity and interaction success have distinct relations in peer and non-peer interactions. Together, these analyses provide the first direct test of relations between neural similarity and interaction success in day-to-day interactions in autistic and non-autistic youth.

## Methods

### Participants

Youth aged 11–14 years old were recruited from the Washington, D.C. area to participate in a longitudinal study including an MRI scan session and EMA data collection. Participants were recruited from previous participation in research, outreach at local events, Facebook advertisements, or through the Simons Foundation Powering Autism Research (SPARK). We appreciate obtaining access to recruit participants through the SPARK research match on SFARI Base.

All participants had an IQ greater than 80 as measured by the Kaufman Brief Intelligence Test-2^38^. All participants were screened to ensure that they did not have a history of head injuries, seizures, or any contraindications for MRI participation (e.g. metal in their body). Participants in the autistic (AUT) group had their autism diagnoses confirmed by a licensed clinical psychologist administering the gold-standard Autism Diagnostic Interview-Revised^39^. Participants in the non-autistic (NON-AUT) group were excluded for a history of psychiatric conditions (except for common comorbidities present in autism: anxiety, depression, obsessive–compulsive disorder or attention-deficit/hyperactivity disorder) or first-degree relative with autism or schizophrenia based on parent report. Following exclusions for MRI data quality (see ‘Neural Similarity Data Analysis’ and EMA data completion (see ‘EMA Data Acquisition’), 92 participants (25 autistic participants, 67 non-autistic participants) were included in the following analyses. Additional information on demographics for these 92 participants is provided in Table 1.

**Table 1:** Demographic Information for Participants Included in Analyses.

	Autistic (*n* = 25)	Non-Autistic (*n* = 67)	Full Sample (*n* = 92)
Mean Age at Scan (min–max)	13.4 (12.0–14.8)	13.1 (11.1–15.0)	13.2 (11.1–15.0)
Mean KBIT-2 Overall Score (min–max)	113 (81–135)	115 (93–142)	115 (81–142)
Gender			
Female	2	31	33
Male	22	34	56
Nonbinary/Genderfluid/Other	1	2	3
Race			
Asian	0	2	2
Black	1	8	9
Did Not Wish to Disclose	0	1	1
More than One Race	2	14	16
White	22	42	64
Ethnicity			
Hispanic/Latino	1	9	10
Not Hispanic/Latino	24	58	82
Highest Parental Education			
Some College/AA Degree	3	4	7
College Degree	6	12	18
Some Graduate School	3	4	7
Post-Graduate Degree	13	47	60
Household Income			
Less than $66,000	2	5	7
$66,000-$100,000	5	5	10
$100,000-$160,000	7	16	23
Over $160,000	11	39	50
Prefer Not to Disclose	0	2	2

As part of the study, participants completed an MRI scan (see ‘Neural Similarity Protocol and Image Acquisition’). Following the MRI scan, participants completed a 10-day EMA protocol (see ‘EMA Data Acquisition’) to report on the success of their day-to-day peer interactions. All procedures were approved by the University of Maryland Institutional Review Board and performed in accordance with relevant guidelines and regulations. Participants and their parents provided informed assent and consent.

### Neural similarity protocol and image acquisition

The scanning protocol consisted of a functional social reward task (data not presented here) and a video viewing paradigm. For the video viewing, youth individually watched a series of short 3–6 min video clips with sound in the scanner, split across three runs each containing two video clips (Supplemental Table S1). Visual stimuli were viewed by participants on a head-coil-mounted mirror, and audio stimuli were presented through earbuds or headphones. Audio volume was tested prior to the scan onset and during test sequences to ensure participants were comfortable and could hear the stimuli by repeating back an example phrase.

Clips were selected to maximize individual differences in interpretation, such as clips that could be interpreted as cloying or sweet, or clips in which participants may focus their attention in different areas (e.g. space shuttle launch vs. astronaut talking), in accordance with previous studies using neural similarity to predict social interaction outcomes^23,40^. After watching the clips in the scanner, youth reported their enjoyment of the clips and reported whether they had seen each clip before.

MRI data were acquired with a 32-channel head coil on a 3 T Siemens Prisma Fit scanner. Participants completed 3 runs of functional data acquisition for the video viewing paradigm (66 interleaved axial slices, multiband factor = 6, voxel size = 2.2 × 2.2x2.2 mm, TR = 1250 ms, echo time = 39.4 ms, flip angle = 90 degrees, pixel matrix = 96 × 96, 399–458 volumes depending on run) and 1 structural scan (T1-weighted, MPRAGE pulse sequence, 192 continuous sagittal slices, voxel size = 0.45 × 0.45x0.9 mm, TR = 1900 ms, echo time = 2.32 ms, flip angle = 9 degrees, pixel matrix = 512 × 512).

### Neural similarity data analysis

Data were pre-processed using a robust preprocessing pipeline, fMRIPrep v20.2.6^41^, with full details provided in Supplemental Information. Briefly, anatomical images were segmented and normalized to MNI space. Functional images were corrected for susceptibility distortion using field maps, warped to the normalized anatomical image, and slice-time corrected. Independent components analysis (ICA-AROMA) was performed to remove motion artifact^42^, and data were spatially smoothed with a 6 mm full width half maximum Gaussian kernel. Functional data were masked to subject-specific functional masks and intensity normalized to a mean of 100 using 3dcalc in AFNI^43^. Then, functional data were regressed for 6 demeaned motion parameters, mean FD, csf (mean and derivative), white matter (mean and derivative), and censored volumes (censored at 1 mm, see below). In accordance with recommendations for processing naturalistic viewing MRI data^17^, 20 volumes were removed from the beginning of each run and 3–4 volumes were removed from the end of each run. Runs were masked to a union mask across the three runs and concatenated for extraction of region of interest (ROI) time series.

Runs with greater than 0.5 mm mean framewise displacement were excluded. Volumes with greater than 1 mm of displacement between volumes were censored, and runs with greater than 20% of volumes censored were also excluded. Runs were excluded if the participant fell asleep or if there were any issues with audio during the scan acquisition that interfered with the participant’s ability to hear the clips. MRI data were also excluded for artifacts or abnormalities based on visual inspection of MRI data. Participants with less than two usable runs were excluded from further analyses (*n* = 7 AUT, *n* = 5 NON-AUT). 12 participants (*n* = 5 AUT, *n* = 7 NON-AUT) were included in analyses with 2 usable runs and 80 participants (*n* = 20 AUT, *n* = 60 NON-AUT) were included in analyses with 3 usable runs. Autistic and non-autistic groups did not differ in mean framewise displacement (*t*(90) = 1.00, *p* = 0.32).

**Fig. 1:**
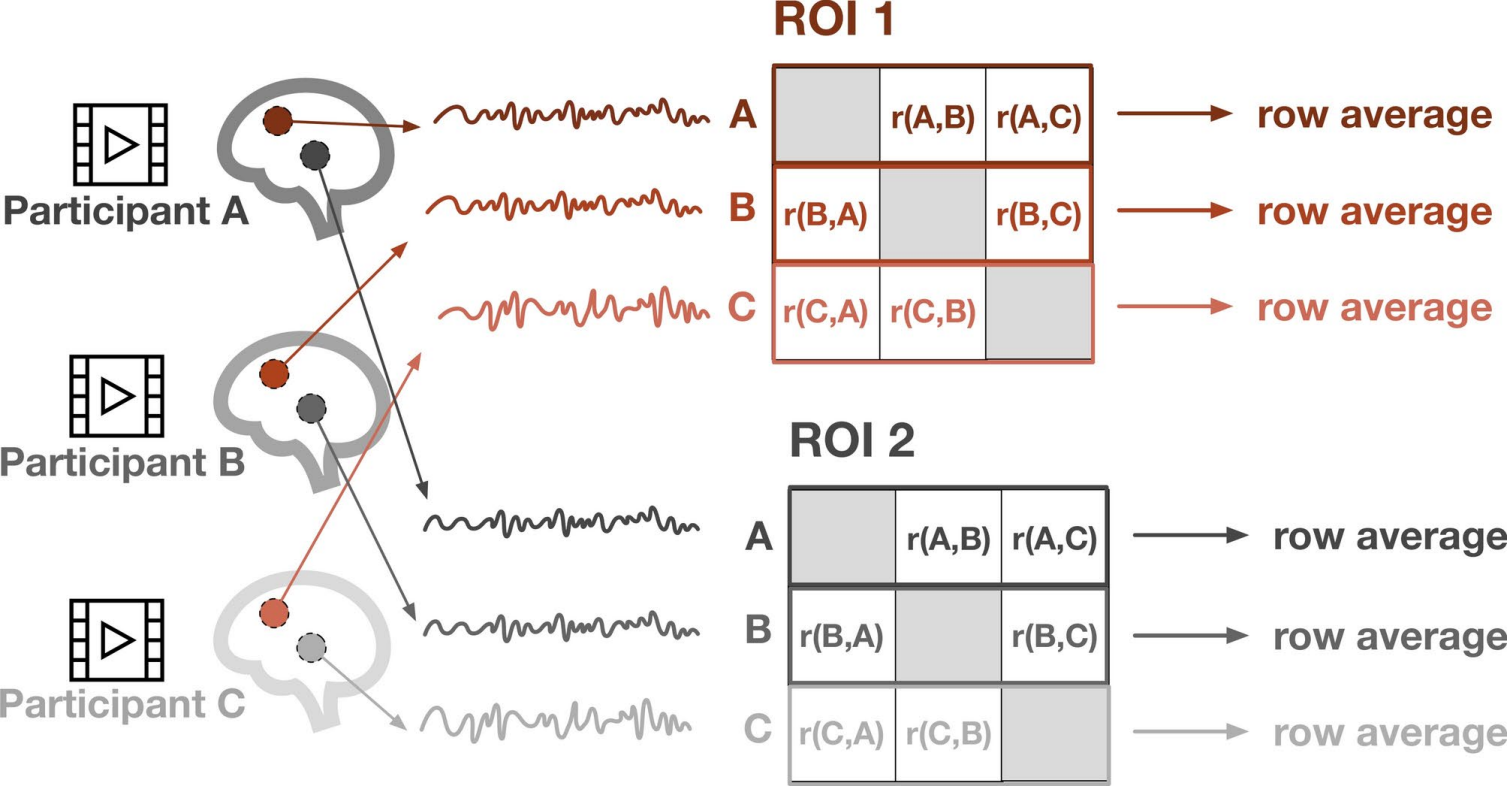
Calculation of neural similarity. Participant-level time series were extracted from functionally derived mentalizing and reward-related ROIs. Within each ROI, time series were correlated across all participants, then Fisher’s *z*-transformed. Then a single value of neural similarity per participant was obtained by averaging all Fisher’s *z* values containing that participant (i.e. the row average).

To calculate neural similarity, average signal was extracted for a set of twelve ROIs. The coordinates for these twelve ROIs were derived from previous work that used automated meta-analysis of neuroimaging literature (Neurosynth^44^), with the search terms for ‘mentalizing’ and ‘reward’^45,46^. ROIs are listed in Table 2. Mentalizing and reward-related regions were chosen based on previous evidence linking neural similarity in these regions to social-interaction-related processes^23,40,47^. Then, each ROI’s BOLD time series was correlated pairwise between all participants using Pearson’s correlation. The correlation values were Fisher’s *z*-transformed. A single neural similarity value for each participant for each ROI was obtained by averaging all Fisher z-transformed correlations including that participant together to obtain that participant’s mean neural similarity to the group (Fig. 1). There were no significant relations between neural similarity and mean framewise displacement in any of the mentalizing and reward-related ROIs.

**Table 2:** List of Twelve Mentalizing- and Reward-Related ROIs.

Name	Abbreviation	MNI Coordinates		
		x	y	z
Anterior cingulate cortex	ACC	2	32	16
Amygdala	AMY			
right	rAMY	24	-2	-18
left	lAMY	-20	-2	-14
Dorsomedial prefrontal cortex	dmPFC	0	53	30
Left anterior temporal lobe	lATL	-53	-12	-16
Left orbitofrontal cortex	lOFC	-22	36	-14
Left temporo-parietal junction	lTPJ	-48	-56	23
Precuneus	Precuneus	0	-54	44
Right anterior temporal lobe	rATL	53	-12	-16
Right temporo-parietal junction	rTPJ	48	-56	23
Right ventrofrontal cortex	rVFC	2	58	-8
Ventromedial prefrontal cortex	vmPFC	0	48	-18
Ventral striatum	VS	± 12	10	-8

Note: Left and right amygdala ROIs were combined to a bilateral amygdala ROI and left and right ventral striatum ROIs were combined to a bilateral ventral striatum ROI because there were no hypotheses about lateralization in these regions. ROIs were extracted as spheres centered around the coordinates (r = 5 mm).

### EMA data acquisition

Participants were enrolled in a ten-day EMA protocol beginning approximately three days after MRI scan completion. During enrollment, youth were given a visual and verbal demonstration of how to complete the prompts, including the study’s definition of a social interaction, and had an opportunity to ask questions with a study experimenter. Participants received 4–5 EMA prompts every day for ten days on a cellphone, with a link to complete a survey. As part of the EMA protocol, participants were asked if they were currently in a social interaction or had been in one within the prompt window, to specify their interaction partners, to respond how ‘close or connected’ they felt to each of their interaction partners (scale of 0-not close at all, to 100-extremely close), and to respond how they felt the interaction went (scale of 0-very bad to 100-very good). See Supplemental Information for additional details on prompt windows and question format, descriptive statistics for EMA variables (Supplemental Table S28), and correlations and distributions of EMA variables (Supplemental Figure S2).

Social interaction closeness for each social interaction reported by a participant was used as a continuous variable and calculated as the average closeness reported by the participant across each of the interaction partners in that interaction. Social interactions were designated as a ‘peer’ interaction if the participant selected that their interaction included a person or people in the following categories: close friend, other friend, classmate/teammate, someone they were dating, or ‘other’ response that could be coded as one of the above (e.g. bandmate). In addition to the peer interaction partner, these ‘peer’ interactions could also have non-peer interaction partners as well. All other social interactions were designated as ‘non-peer’ interactions.

Participants were excluded from further EMA analysis for not having any responses that reported a social interaction (*n* = 1 AUT). Participants that were included in analyses had varying numbers of responses completed that described a social interaction (*n* = 3–53 responses per participant for a total of 2280 responses).

### EMA analysis

We first investigated whether the social partner (peer/non-peer) and group (autistic/non-autistic) statistically interacted to predict social interaction success in the EMA data. A multilevel model was constructed treating EMA observations as nested within individuals. Intercepts were modeled as randomly varying across individuals. Interaction success was used as the outcome variable, and social partner (1 = peer, 0 = non-peer) was incorporated as both a person-level mean and a social-interaction-level predictor centered on each person’s mean to examine effects of social partner at between-person and within-person levels, respectively^48^. Two statistical interaction terms were modeled, one between the person-mean-centered social partner variable and group (autistic = 0, non-autistic = 1), and the other between the person-mean social partner variable and group. These statistical interactions test whether the between-person and within-person effects of social partner depend on autism diagnosis. Age and gender were included as covariates. We also conducted a resampling analysis to test this model in a balanced autistic/non-autistic sample. The results are consistent with the results presented in the main text (see Supplemental Information).

### Neural similarity-to-EMA analysis

We next investigated relations between neural similarity and interaction success in the EMA data. The hypotheses and analysis plan were pre-registered on the Open Science Framework (https://osf.io/g6mv4) following data collection but prior to the start of data analysis. Amendments to the pre-registered plans are detailed in Supplemental Information.

First, we tested relations between neural similarity and interaction success (pre-registered hypothesis 1). A multilevel model was constructed treating EMA observations as nested within individuals. Intercepts were modeled as randomly varying across individuals. Interaction success was the outcome variable, neural similarity was the predictor of interest, and group (autistic/non-autistic), gender, and age were included as covariates. Twelve models were evaluated, one for each of the twelve ROIs, and FDR correction was performed across the twelve models to account for multiple comparisons. Post-hoc exploratory analyses were also conducted averaging neural similarity across mentalizing ROIs and reward ROIs. That is, neural similarity values for each of the seven mentalizing-related ROIs (dmPFC, lATL, lTPJ, precuneus, rATL, rTPJ, vmPFC) were averaged together to obtain one ‘mentalizing’ neural similarity value, and neural similarity values for each of the seven reward-related ROIs(ACC, lAMY, lOFC, lVS, rAMY, rVFC, rVS) were averaged together to obtain one ‘reward’ neural similarity value.

To follow up, we tested whether the relations between neural similarity and interaction success more specifically followed an Anna Karenina model^24^, such that individuals with better interaction success were all neurally alike, while individuals who reported worse interaction success were all neurally idiosyncratic (pre-registered hypothesis 2). We implemented these models as multilevel models with crossed random effects, with neural similarity between any given pair of participants in a given ROI as the outcome, the mean of the pair of participants’ averaged interaction success ratings as a predictor, and random intercepts for each participant in the pair^49^. In line with recommendations for using models with crossed random effects to analyze intersubject correlation data, we performed analyses on fully crossed data (8372 correlations) and performed manual correction on the *t*-statistic and degrees of freedom to account for the 4186 unique correlations^49^. We also tested whether these relations held above and beyond youth-reported similarity on video stimuli preferences (pre-registered hypothesis 2a). For these models, an additional predictor of pairwise video preference similarity between pairs of participants was calculated as 1-(Euclidean distance of the two participants’ responses across the six video enjoyment self-report questions/maximum Euclidean distance), in line with previous work^22^.

Next, we tested whether youth-perceived closeness interacted with neural similarity in predicting interaction success (pre-registered hypothesis 1a). A multilevel model was constructed treating EMA observations as nested within individuals, and intercepts were modeled as randomly varying across individuals. The model failed to converge when a random slope was included to allow the closeness variable slope to vary across individuals, but the model converged with the slope modeled as fixed. Therefore, the slope was modeled as fixed. Closeness was incorporated as a person-mean and person-mean-centered predictor to distinguish between relations at the between-person and within-person levels, respectively^48^. Two statistical interaction terms were modeled, one between the person-mean-centered closeness variable and neural similarity, and the other between the person-mean closeness variable and neural similarity. Age, gender, and group were included as covariates. Again, twelve models were evaluated, one for each of the twelve ROIs, and FDR correction was performed across the statistical interaction coefficients of interest for the twelve models to account for multiple comparisons. Post-hoc exploratory models were conducted averaging neural similarity across mentalizing ROIs and across reward ROIs, which were treated as independent tests.

Finally, we performed an exploratory test of whether the social interaction partner (peer/non-peer) interacted with neural similarity in predicting interaction success. A multilevel model was created with EMA observations nested within individuals. Intercepts were modeled as randomly varying across individuals, and a random slope was included to allow the social partner variable slope to vary across individuals. As before, the social interaction partner (0 = non-peer, 1 = peer) was incorporated as a person-mean and person-mean-centered variable, and both statistical interactions with neural similarity were modeled. Age, gender, and group were included as covariates. Models were evaluated for twelve ROIs and FDR correction was performed. Again, post-hoc exploratory models were conducted averaging neural similarity across mentalizing ROIs and across reward ROIs, which were treated as independent tests.

Between-group (autistic vs. non-autistic) differences in neural similarity across the Shen parcellation and 12 ROIs were also tested. No parcels or regions survived correction for multiple comparisons (see Supplemental Information). Means and standard deviations of neural similarity by group are provided in Supplemental Information (Supplemental Table S27).

In line with the preregistration, several additional exploratory analyses were also conducted, and results are detailed in Supplemental Information. Specifically, hypotheses were tested using a 268 parcel whole-brain parcellation^50^. Hypothesis 1a was also tested using closeness defined through a forced-choice question in which social interactions were categorized as either ‘close’ or ‘not close’. Hypotheses were also tested within the non-autistic group and within the autistic group.

Finally, hypotheses were tested using neural similarity calculated for each participant relative to the non-autistic group. These results were conceptually identical to the results presented in the main text.

## Results

### Self-reported day-to-day interaction success

**Fig. 2:**
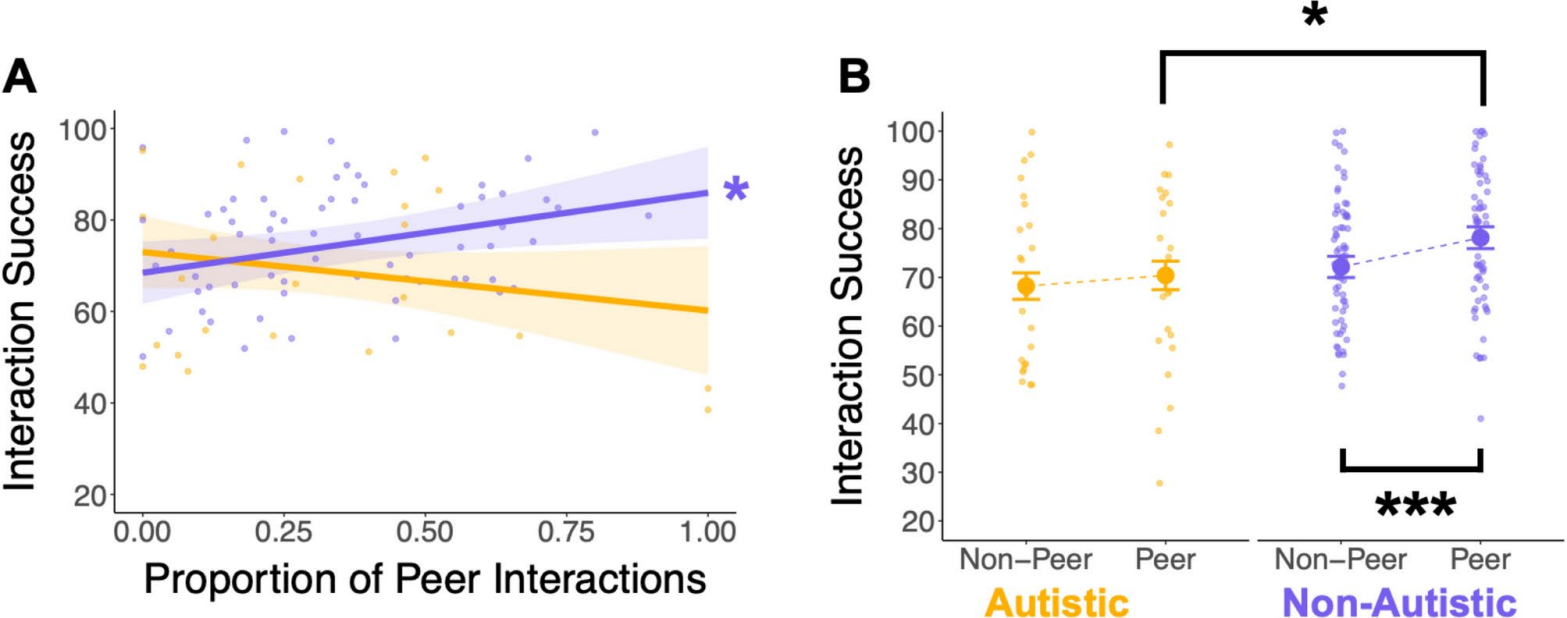
Peer interactions differentially contribute to interaction success for autistic and non-autistic youth. A) Non-autistic youth reported significantly more interaction success as they reported a greater proportion of their interactions being with peers (*, *p* = 0.02), while autistic youth reported a non-significant decline in interaction success as a greater proportion of their interactions were with peers. B) Non-autistic youth report significantly greater success during peer interactions compared to non-peer interactions (***, *p* < 0.001) and also report greater success during peer interactions compared to autistic youth (*, p = 0.03). The groups did not differ in interaction success during non-peer interactions. Large circles indicate group means and error bars depict ± standard error of means. Small circles indicate participant-level means.

We first examined whether group (autistic/non-autistic), the social interaction partner (peer/non-peer), and their statistical interaction significantly predicted youth-reported interaction success, controlling for age and gender. There was a marginally significant main effect of group on interaction success (*B* = 5.83, *t*(89.62) = 1.77, *p* = 0.08), and there was a significant statistical interaction between individuals’ mean proportion of peer interactions and group in predicting interaction success (*B* = 30.27, *t*(96.67) = 2.48, *p* = 0.01; Fig. 2A). For autistic individuals, there was a non-significant negative relation between proportion of peer interactions and interaction success, such that reporting a higher proportion of interactions with a peer partner was associated with a non-significant decline in interaction success (*B* = -12.79, *t*(103.23) = -1.32, *p* = 0.19). By contrast, for non-autistic individuals, reporting a higher proportion of peer interactions was associated with a significant increase in interaction success (*B* = 17.48, *t*(85.62) = 2.36, *p* = 0.02).

There was a marginal statistical interaction for the within-person peer interaction variable and group (*B* = 3.79, *t*(2785.94) = 1.90, *p* = 0.06; Fig. 2B). Non-autistic youth reported significantly more success in peer interactions compared to non-peer interactions (*B* = 5.98, *t*(2785.94) = 7.04, *p* < 0.001), while autistic youth did not report significantly different interaction success between peer and non-peer interactions (*B* = 2.19, *t*(2785.94) = 1.21, *p* = 0.22). Non-autistic youth also reported significantly greater success during peer interactions compared to autistic youth (*B* = 7.73, *t*(119.83) = 2.18, *p* = 0.03), while there were no group differences in reported success in non-peer interactions (*B* = 3.94, *t*(94.21) = 1.18, *p* = 0.24).

### Links between neural similarity and self-reported day-to-day interaction success

Next, relations between neural similarity and self-reported day-to-day interaction success were evaluated in the full sample of autistic and non-autistic youth. There were no significant links between neural similarity and day-to-day interaction success following FDR-correction (all *p*s > 0.10; Supplemental Table S2). There were also no significant links between neural similarity and day-to-day interaction success when neural similarity was averaged across mentalizing-related ROIs or averaged across reward-related ROIs. Follow-up exploratory analyses were performed to determine if there were any significant links between neural similarity and interaction success in each of the six videos. There were no significant relations in any of the videos (Supplemental Tables S3-S8).

We performed follow-up analyses to determine whether any of the brain regions displayed an Anna Karenina relationship between neural activity and interaction success, such that individuals who have better day-to-day interaction success are all neurally alike, while individuals who have worse day-to-day interaction success are all neurally idiosyncratic (different from all others in sample). No ROIs significantly followed this model (Supplemental Table S9), and no ROIs significantly followed this model when accounting for similarity in self-reported video preferences (Supplemental Table S10).

**Fig. 3:**
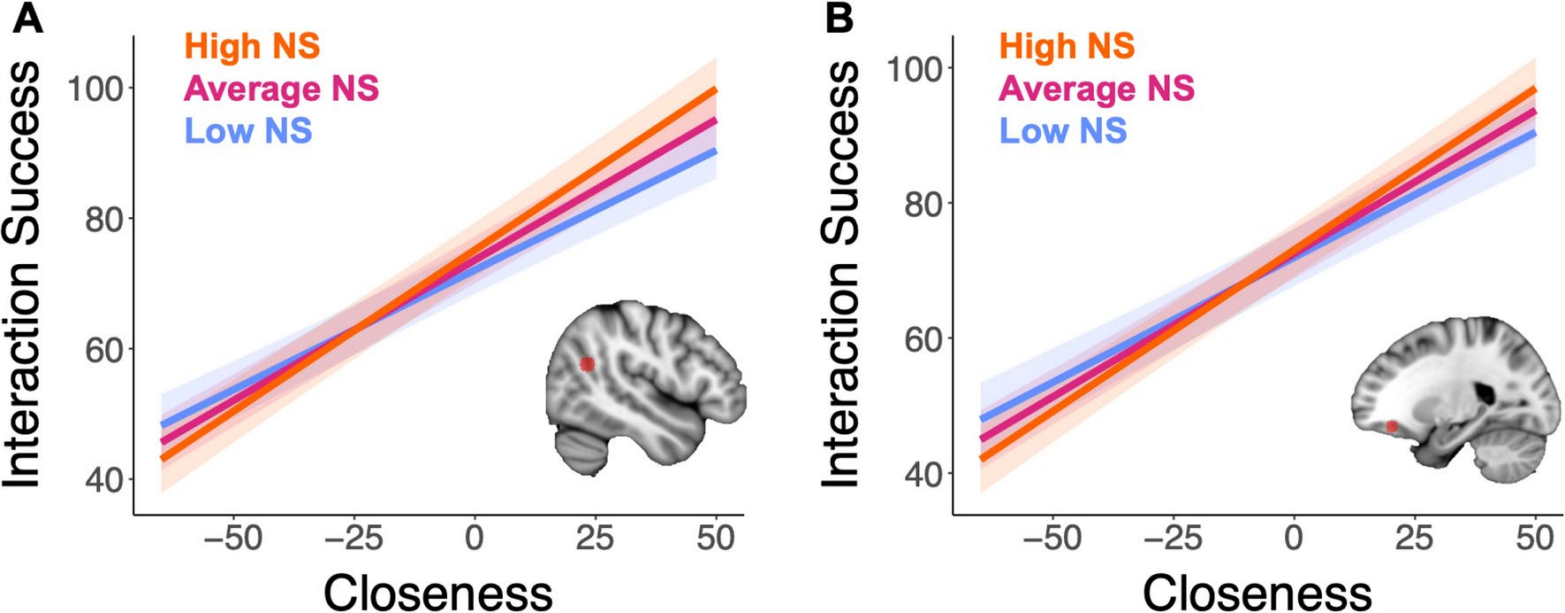
Closeness and neural similarity interact to predict interaction success in rTPJ and lOFC. A) Similarity in rTPJ activity statistically interacted with youth-reported closeness to predict social interaction success. Individuals with higher neural similarity in rTPJ had a stronger relation between interaction success and perceived closeness than individuals with weaker neural similarity in rTPJ. Put another way, the relation between neural similarity and interaction success was stronger in social interactions rated as closer relative to a participant’s mean. B) Similarity in lOFC activity statistically interacted with youth-reported closeness to predict interaction success. Individuals with higher neural similarity in lOFC had a stronger relation between interaction success and perceived closeness than individuals with weaker neural similarity in lOFC. Put another way, the relation between neural similarity and interaction success was stronger in social interactions rated as closer relative to a participant’s mean. High NS = high neural similarity (1 standard deviation above mean), Average NS = mean neural similarity, Low NS = low neural similarity (1 standard deviation below mean).

Next, we tested whether youth perceptions of closeness and neural similarity interacted to predict interaction success (Supplemental Table S11). There was a significant statistical interaction between person-centered closeness and neural similarity in predicting interaction success that survived FDR correction in both the rTPJ (*B* = 1.59, *t*(2405.2) = 3.97, *p*_corrected_ < 0.001; Fig. 3A) and lOFC (*B* = 2.69, *t*(2405.4) = 2.97, *p*_corrected_ = 0.02; Fig. 3B). Probing the rTPJ interaction showed that there was a stronger relation between neural similarity in the rTPJ and interaction success for closer interactions (1 standard deviation above the mean; *B* = 65.95, *t*(106.37) = 3.08, *p* = 0.003), while there was a non-significant relation between neural similarity in the rTPJ and interaction success for less close interactions (1 standard deviation below the mean; *B* = 10.65, *t*(106.37) = 0.50, *p* = 0.62). Put another way, there was a stronger relation between ratings of closeness and interaction success for individuals with higher rTPJ neural similarity (*B* = 0.50, *t*(2405.2) = 20.14, *p* < 0.001), while the relation weakened, though remained significant, for individuals with lower rTPJ neural similarity (*B* = 0.36, *t*(2405.2) = 14.64, *p* < 0.001; Fig. 3A).

Probing the lOFC interaction demonstrated a similar pattern (Fig. 3B); there was a non-significant positive relation between neural similarity and interaction success for closer interactions (*B* = 74.40, *t*(106.90) = 1.58, *p* = 0.12) and a non-significant negative relation for less close interactions (*B* = -19.12, *t*(106.90) = -0.41, *p* = 0.69). When exploring the statistical interaction effect for high and low levels of neural similarity, for individuals with higher lOFC similarity, there was a stronger relation between ratings of closeness and interaction success (*B* = 0.48, *t*(2405.4) = 19.90, *p* < 0.001) while the relation was weakened for those with lower lOFC similarity (*B* = 0.37, *t*(2405.4) = 13.56, *p* < 0.001).

We also tested whether youth perceptions of closeness and neural similarity statistically interacted to predict social interaction success in an exploratory follow-up analysis when neural similarity was averaged across mentalizing-related ROIs and averaged across reward-related ROIs. Average mentalizing neural similarity significantly interacted with closeness to predict interaction success (*B* = 1.25, *t*(2405.3) = 2.00, *p* = 0.045). This relation was similar to the relation observed in the rTPJ ROI, such that there was a stronger relation between ratings of closeness and interaction success for individuals with higher neural similarity in mentalizing ROIs (*B* = 0.46, *t*(2405.3) = 19.42, *p* < 0.001), while the relation weakened, though remained significant, for individuals with lower neural similarity in mentalizing ROIs (*B* = 0.39, *t*(2405.3) = 15.30, *p* < 0.001). Average neural similarity in reward-related ROIs did not significantly interact with closeness to predict interaction success.

Follow-up exploratory analyses were performed to determine whether evidence for a statistical interaction between neural similarity and person-mean-centered interaction closeness was present in each of the six videos, which differed in content and could therefore give a preliminary understanding of what domains of processing may underlie these effects (Supplemental Tables S12-S17). Significant evidence for a statistical interaction between rTPJ similarity and closeness in predicting interaction success was found in the Partly Cloudy, Astronauts in Space, and Office Music Video stimuli (Supplemental Tables S12, S14, and S16), while significant evidence for a statistical interaction between lOFC similarity and closeness in predicting interaction success was found in the Partly Cloudy, Astronauts in Space, and Superhero Music Video stimuli (Supplemental Tables S12, S14, and S15). Results held when full scale IQ was included as a covariate. Results also held for exploratory within-group analyses of just the autistic group (Supplemental Table S26).

**Fig. 4:**
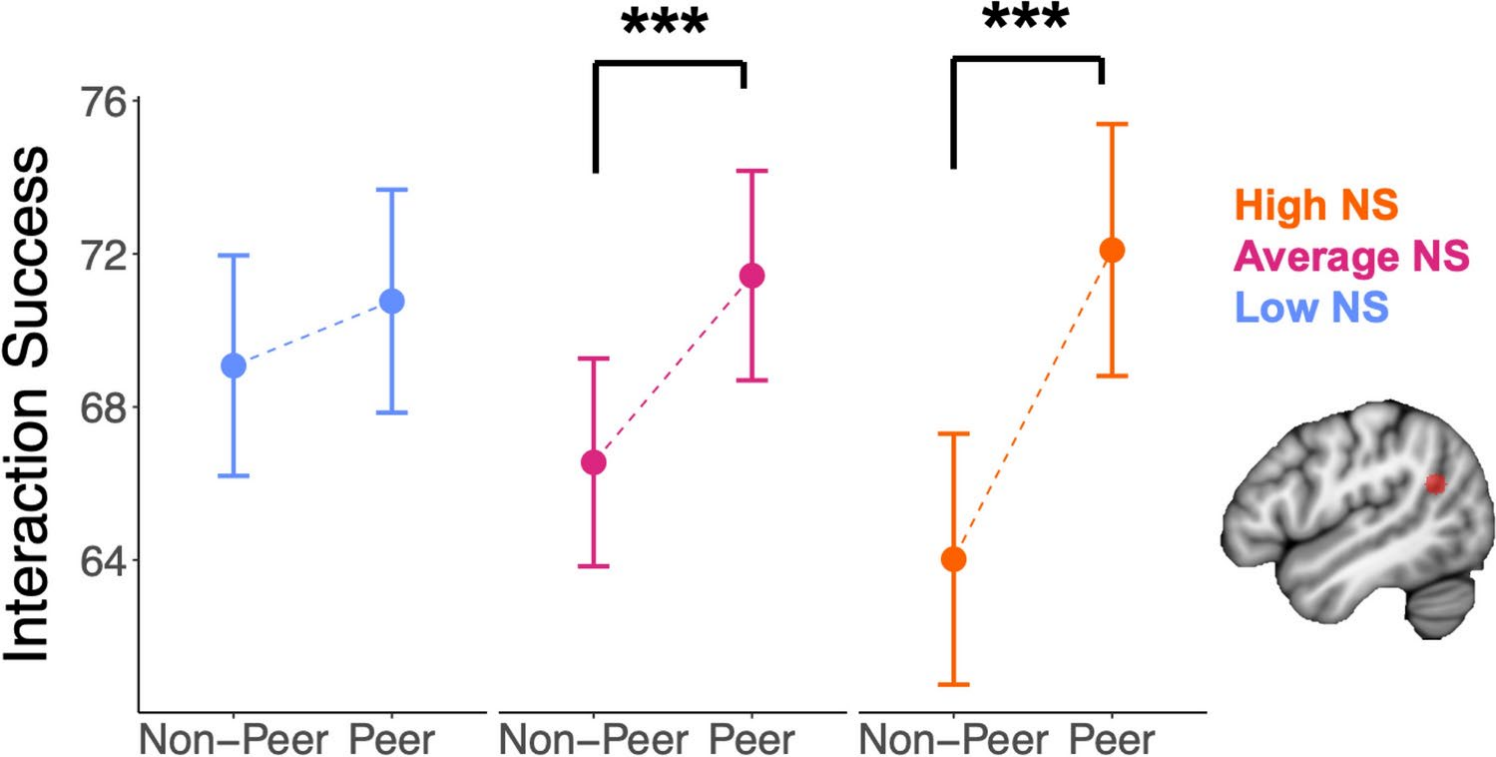
Social partner (peer/non-peer) and neural similarity marginally interact to predict interaction success in lTPJ. There was a significant difference between peer and non-peer interaction success for individuals with higher lTPJ neural similarity (1 standard deviation above the mean; ***, *p* < 0.001), and average lTPJ neural similarity (***, *p* < 0.001), while there was no significant difference between peer and non-peer interaction success for individuals with lower lTPJ neural similarity (1 standard deviation below the mean). Put another way, there was a positive relation between neural similarity in the lTPJ and interaction success for peer interactions, while there was a negative relation between neural similarity in the lTPJ and interaction success for non-peer interactions. Error bars indicate ± standard error.

Finally, we tested whether the social partner (i.e., peer or non-peer) statistically interacted with neural similarity to predict interaction success (Supplemental Table S18). There was a marginally significant statistical interaction between the person-mean-centered social partner variable (0 = non-peer, 1 = peer) and neural similarity in predicting interaction success after FDR correction in the lTPJ (*B* = 74.01, *t*(71.04) = 2.76, *p*_corrected_ = 0.09). Probing the lTPJ interaction showed that there was a non-significant positive relation between neural similarity in the lTPJ and interaction success for peer interactions (*B* = 15.47, *t*(80.08) = 0.45, *p* = 0.65), while there was a marginally significant negative relation between neural similarity in the lTPJ and interaction success for non-peer interactions (*B* = -58.54, *t*(84.09) = -1.72, *p* = 0.09). Put another way, there was a significant difference between peer and non-peer interaction success for individuals with higher lTPJ neural similarity (1 standard deviation above the mean; *B* = 8.08, *t*(66.29) = 5.05, *p* < 0.001), while there was no significant difference between peer and non-peer interaction success for individuals with lower lTPJ neural similarity (1 standard deviation below the mean; *B* = 1.69, *t*(76.60) = 1.00, *p* = 0.32; Fig. 4).

We also tested whether social partner (peer/non-peer) and neural similarity interacted to predict interaction success in an exploratory follow-up analysis when neural similarity was averaged across mentalizing-related ROIs and averaged across reward-related ROIs. Average neural similarity in mentalizing-related ROIs significantly interacted with social partner to predict interaction success (*B* = 97.23, *t*(75.60) = 2.23, *p* = 0.03). This relation was similar to the relation observed in the lTPJ ROI, such that there was a significant difference between peer and non-peer interaction success for individuals with higher neural similarity across mentalizing-related ROIs (1 standard deviation above the mean; *B* = 7.65, *t*(67.31) = 4.64, *p* < 0.001), while there was no significant difference between peer and non-peer interaction success for individuals with lower neural similarity across mentalizing-related ROIs (1 standard deviation below the mean; *B* = 2.38, *t*(79.73) = 1.41, *p* = 0.16). Average neural similarity in reward-related ROIs did not significantly interact with social partner to predict interaction success.

Follow-up exploratory analyses were performed to determine whether evidence for a statistical interaction between neural similarity and social partner was present in each of the six videos (Supplemental Tables S19-S24). Significant evidence for a statistical interaction between lTPJ similarity and social partner in predicting interaction success was found in the Partly Cloudy video (Supplemental Table S19). Results held when full scale IQ was included as a covariate.

## Discussion

In the first study to directly examine relations between autistic and non-autistic youth’s neural similarity and their day-to-day interaction success, we found no significant relations between neural similarity in mentalizing and reward-related brain regions and youth-reported social interaction success. However, we found that relations between neural similarity and youth-reported interaction success significantly differed depending on interaction closeness and differed depending on whether or not the interaction was with a peer. Specifically, we found that youth-reported interaction closeness significantly interacted with neural similarity in the mentalizing network as a whole in an exploratory network-wide analysis, as well as significantly interacted with neural similarity in the rTPJ and lOFC to predict interaction success. Interestingly, these relations were in the opposite direction as hypothesized; we found that there was a stronger relation between neural similarity in these regions and interaction success for closer interactions, while there was a weaker relation between neural similarity and interaction success for less close interactions. Put another way, individuals who were more neurally similar had a stronger relation between closeness and interaction success, while individuals who were less neurally similar had a weaker relation between closeness and interactions success.

A peer being involved in the interaction was also a factor in interaction success. Social partner (peer/non-peer) significantly interacted with neural similarity in the mentalizing network in an exploratory analysis averaged across mentalizing ROIs and specifically marginally interacted with the lTPJ region within that network to predict interaction success, with positive relations between neural similarity and interaction success for peer interactions and negative relations for non-peer interactions. This uniqueness of peer interactions was further highlighted by the influence of peer interactions on interaction success for autistic and non-autistic youth. While non-autistic youth reported significantly better interactions if they had a higher proportion of peer interactions, autistic youth did not. Together, these findings highlight the involvement of similarity to one’s peers in mentalizing and reward processing in youth’s day-to-day interaction enjoyment and emphasize the ways in which peers influence interaction success for autistic and non-autistic youth in early adolescence.

### Absence of relations between neural similarity and interaction success

Contrary to our hypothesis, we did not find a significant main effect between neural similarity in mentalizing or reward-related brain regions and youth-reported day-to-day interaction success. We did not find these links either in models testing linear relations between neural similarity and interaction success (hypothesis 1) or in models testing whether the relations followed an Anna Karenina model^24^, such that individuals with better interaction success were all neurally alike, while individuals who reported worse interaction success were all neurally idiosyncratic (hypothesis 2). Previous work has shown links between neural similarity in social brain regions and social outcomes like loneliness and popularity^22,23^. However, with respect to day-to-day interaction success, factors that predict day-to-day interaction success may differ across the heterogeneity of interactions experienced in daily life: for example, interactions with close others compared to less close others or interactions with and without peers. Our results support this interpretation, suggesting that neural similarity may not predict day-to-day interaction success across all interactions, but may play more of a role in specific interactions by significantly moderating relations between other factors (i.e. close as opposed to non-close, peer as opposed to non-peer) and interaction success.

One limitation of our approach is that we are not able to directly measure neural similarity between individuals and each of their day-to-day interaction partners. Instead, we rely on an individual’s neural similarity to other individuals in the study group as an estimate of neural similarity that an individual has to others their age. In contrast, other previous work characterizing relations between neural similarity and social outcomes directly examined similarity between the two interaction partners^47^, or between individuals who were within each other’s social networks, such as students in the same dorm or program^22,23,40^, as opposed to the participants in our study who largely lived in different neighborhoods and attended different schools across the greater DC area. Therefore, the neural similarity metric may not capture shared perspectives with others in participant’s day-to-day social world in the same way as previous studies. Future work will assess relations between neural similarity and interaction success for interacting autistic and non-autistic youth where neural activity for both individuals is measured. Additionally, while averaging signal across regions of interest has been previously used to uncover individual differences in relevant social outcomes^22–24,40^, use of multivoxel pattern analysis approaches^51^ could be incorporated into future research to shed additional light on the ways neural pattern similarity relates to interaction success.

In the present study, interaction success was measured as a self-report by asking youth how well they felt the interaction went. This self-report could capture aspects of both ‘objective’ success, such as whether communicative and affiliative goals were achieved, as well as the youth’s perception of their interaction abilities and subjective enjoyment of the experience. Neural similarity may relate differently to these different aspects of interaction success. Previous research investigating links between neural similarity and interaction success in partners that interacted found that partners who were more neurally similar had more successful interactions when success was measured as an outcome in a communicative game^47^. However, neural similarity did not significantly predict rapport; instead, individuals’ self-reported perceived similarity best predicted rapport^47^. Both subjective and more objective ratings of conversational quality are relevant to understanding and supporting autistic and non-autistic youth in social interactions, as both contribute to well-being, mental health, and navigating the social world^52^. Future work could attempt to disentangle these aspects of interaction success and relations to neural similarity in autistic and non-autistic youth with the addition of interaction partner and/or observer ratings of interaction success.

### Neural similarity in rTPJ and lOFC interacts with closeness to predict interaction success

While there was no main effect of neural similarity on interaction success, neural similarity did significantly interact with closeness to predict interaction success. Contrary to our hypothesis, we found that for less close interactions there was a weaker relation between neural similarity and interaction success, while for closer interactions there was a stronger relation between neural similarity and interaction success. Put another way, more neurally similar individuals have stronger links between closeness and interaction success while less neurally similar individuals have a weaker link. This interaction held in both the rTPJ and lOFC, regions involved in mentalizing and reward-related processes respectively, and further held across averaged mentalizing-related ROIs in an exploratory analysis, with other mentalizing-related ROIs demonstrating non-significant trends in the same direction. While previous work has shown links between perceived closeness and aspects of interaction success like positive affect^53^, this is the first work showing that the strength of this relation may vary based on neural similarity.

The rTPJ is a key brain region involved in considering others’ mental states across development^54–56^. The rTPJ may therefore play a moderating role in relations between interaction success and closeness because this region is involved in representing others’ mental states. This effect may be more robust in the mentalizing heavy videos such as Partly Cloudy and a music video depicting office bullying. Having higher rTPJ neural similarity may mean that these individuals have more shared perspective taking, such that closeness for these individuals takes on more of a mental state meaning and is more tightly linked to fluctuations in interaction success. In contrast, having lower rTPJ neural similarity may mean that these individuals have reduced shared perspective taking, which could further relate to either (a) more heterogeneous conceptualization of closeness, including interpretations of closeness that are less reliant on mental states and/or (b) more heterogeneous conceptualization of interaction success, with factors other than closeness contributing to interaction success as well. As our relations held controlling for IQ, these results seem to not reflect differences in general cognition. Instead, these results may reflect idiosyncrasy in mentalizing processes related to understanding closeness and success. Past work has linked idiosyncrasy in mentalizing network connectivity with idiosyncratic verbal interpretations of mentalizing movie events^57^. Follow up research could support this interpretation by more directly probing the factors that contribute to idiosyncratic understanding of interaction success and/or closeness in these participants. While the relation was seen primarily for mentalizing heavy videos, this interpretation that the rTPJ’s role in moderating interaction closeness and success is driven by response to mentalizing events would be strengthened by specifically probing neural response to mentalizing events in these videos, as others have done by coding mentalizing events within the video^58^. Previous work has identified a similar role for rTPJ function in moderating relations between adolescents’ self-reported closeness and positive affect^59^. Adolescents with weaker rTPJ response to a social reward paradigm had stronger relations between closeness and concurrent happiness, but weaker relations between closeness and future happiness, than adolescents with stronger rTPJ response^59^, again providing links between rTPJ activity and perceptions of closeness.

The lOFC was also found to play a significant moderating role between closeness and interaction success. The OFC is a region in the reward network, with multiple meta-analyses linking OFC function to the rewarding value of outcomes^60–62^. Having more shared understanding of reward, especially in videos featuring social interactions like Partly Cloudy and a music video featuring peer victimization and neglect, may mean that neurally similar individuals’ perceptions of closeness may be more imbued with perceptions of value, which in turn more strongly relates to perceptions of interaction success. Having less shared understanding of reward value in social interaction may mean that an individual’s understanding of closeness takes on varied meaning that may not be as tied to reward and is less closely linked to perceptions of success.

### Uniqueness of peer relations in early adolescence

A peer being involved in the interaction contributed differentially to interaction success between autistic and non-autistic individuals, as well as for relations between lTPJ neural similarity and interaction success. Non-autistic youth rated their peer interactions as more successful than their non-peer interactions and experienced a significant boost in reported interaction success when a greater proportion of their interactions were with peers. In contrast, autistic youth rated their peer interactions as less successful than non-autistic youth and experienced a slight, though non-significant, decline in interaction success when a greater proportion of their interactions were with peers. While our results should be viewed as preliminary given the small autistic sample, they highlight the importance of understanding peer relations in autistic youth. Autistic youth report a desire for and interest in interaction with peers and forming friendships with other youth^2,63^. Yet autistic youth also report high rates of negative encounters with peers, such as peer victimization or bullying^64^, with negative impacts on their well-being^5,65^. Therefore, increasing rates of peer interactions for autistic youth may not necessarily reflect increased opportunities for forming the relations and having the positive peer interactions that they desire. These increasing opportunities of peer interaction could instead be leading to increased opportunities for victimization by peers, meaning that these peer interactions are not meeting autistic individuals’ social needs and contributing to their feelings of successful interactions. This highlights the importance of future opportunities to prevent peer victimization in autistic youth, including educating peers on autism acceptance and understanding^66,67^.

Social partner (peer/non-peer) also interacted with neural similarity to predict interaction success. There was a marginally more positive relation between neural similarity in lTPJ and interaction success for peer compared to non-peer interactions, or, phrased another way, youth who were more neurally similar in lTPJ had a greater discrepancy in interaction success between their peer and non-peer interactions. A significant statistical interaction between neural similarity and social partner was also observed when neural similarity was averaged across all mentalizing-related ROIs in an exploratory analysis, which may capture the fact that many mentalizing ROIs (e.g. rTPJ, lATL) non-significantly trended in the same direction, though only lTPJ marginally survived correction. Different relations between neural similarity and interaction success in peer and non-peer relations could be driven by multiple factors. First, our neural similarity measure was calculated relative to other youth in the study, which may mean that our neural similarity metric better captures ‘neural similarity to peers’ and shared understanding with peers than ‘neural similarity to non-peers’. Second, adolescence may represent a period of social reorienting towards peers, with corresponding neural and behavioral changes^68,69^. In the present study, the ROI where this relation was identified was the lTPJ. This region is known for involvement in mentalizing and social interaction^56,70^ and undergoes continued structural and functional development during adolescence^71,72^. As part of this functional development, the left TPJ begins to take on more of a role in mentalizing processes throughout adolescence^54^. This functional development of the lTPJ could account for the specificity of these peer findings to the lTPJ, though the ROI only marginally survived correction and other mentalizing ROIs also non-significantly trended in the same direction. As the video-by-video analysis identified the interaction between neural similarity and peer interactions as specific to the mentalizing-heavy Partly Cloudy video, this further supports the interpretation that similarity in mentalizing processes may play differential roles in peer and non-peer interaction success in adolescence, though future research could more directly test this hypothesis.

### Limitations/future directions

One limitation of the present work is the small size and lack of gender diversity in the autistic sample. Because of the limited size of the autistic sample, the sample was best powered to test relations of neural similarity to interaction success across the full sample, and future research should include larger autistic samples to better test for differences between autistic and non-autistic samples. There are known gender differences in peer interaction in non-autistic adolescents, with boys more likely to have positive peer experiences in larger classmate/teammate groups than girls and girls more likely to report higher quality friendships than boys^73,74^. However, the present study had a largely male autistic sample, which limited our ability to probe gender differences in the autistic sample. Having better representation of female and non-binary autistic adolescents is important because there are gender differences in relation to peer social relationships for autistic girls and boys^75,76^. Future research should prioritize larger and more gender diverse autistic samples.

An additional limitation is that we observed between-participant variability in EMA response rates. All participants were included regardless of their adherence to the protocol and the number of prompts they completed, in line with some recommendations to consider potential bias invoked by excluding participants based on prompt completion^77^. All participants were included because participants who responded to fewer prompts also reported worse interaction success on average, and excluding these participants would have biased the analysis by systematically excluding youth reporting worse interactions. However, the responses included for participants with very few responses may not provide a robust characterization of those participants’ day-to-day experiences of social interaction success.

Future work could also benefit from diverse approaches to capturing similarity across individuals in the sample. In this paper, we calculated neural similarity as individual-to-group by averaging all correlations to other individuals into one neural similarity value. There are other approaches to considering patterns in similarity across individuals in the sample, such as clustering approaches to identify groups within the sample that are similar to each other in neural activity^30,33^, which could then be used to identify if these groups also display similar patterns in their day-to-day interaction success. Future analyses could use these approaches to better identify clusters within the sample. Additionally, our use of an intersubject correlation approach to calculate similarity by averaging signal within ROIs and correlating across these averaged time series may miss information at the voxel level. Multivoxel pattern analysis approaches provide additional information on the patterns of information contained within the ROIs^51^. These approaches may shed additional light on the ways neural pattern similarity relates to interaction success. Dynamic ISC or event-based approaches could further provide additional information on which events or time periods within the stimulus are driving relations between neural similarity and interaction success.

Additional factors such as attention to the stimuli may also impact neural similarity relations. Previous work in autistic and neurotypical individuals has demonstrated a relation between eye movement patterns while watching video stimuli and neural similarity^78^. Future work could incorporate eye-tracking data collection to directly test the role eye movement patterns play in the relations observed in this study.

One limitation of the EMA protocol was that youth could select many interaction partners but only assign one rating to the overall interaction success. Therefore, interactions were coded as ‘peer’ if they involved peers, but they could also have involved other non-peer interaction partners who may have influenced the interaction success rating. Future protocols could collect specific measures of success with respect to each individual with whom the participant interacted. Additionally, the ‘non-peer’ category is diverse and could include parents, siblings, and other family/community members, with these groups potentially playing unique roles in the youth’s life. Future work could more specifically probe these relations with neural similarity and interaction success.

## Conclusion

We demonstrated that peer interactions and perceptions of closeness interact with neural similarity, predominantly within the mentalizing network, to predict youth’s reports of interaction success. We further identified that non-autistic youth report they have more successful interactions with peers, while autistic youth do not report the same ‘peer interaction benefit’. Overall, these findings underscore the role of shared understanding of mental states and reward value in interaction success and closeness for early adolescents and highlight a specific opportunity to improve peer interaction success for autistic youth, further supporting youth well-being.

## Supplementary Information

Supplementary Information.

## Data Availability

The data from consenting participants that support the findings of this study have been uploaded to the National Institute of Mental Health Data Archive (NDA) under collection #3769 and are available upon request at https://nda.nih.gov.
