# Supplementary material for: Neural similarity and interaction success in autistic and non-autistic adolescents
Source: Sci Rep. 2025 Mar 7;15:7996. doi: 10.1038/s41598-025-91176-9 (PMC11889181; doi:10.1038/s41598-025-91176-9)
Supplement: Supplementary file 1 — Supplementary Information. [file 41598_2025_91176_MOESM1_ESM.docx]

**Supplemental Information**

**Stimuli**

*Supplemental Table S1. Description of video clips used for neural similarity data collection*

| Clip Name (run) | Length (s) | Description |
| --- | --- | --- |
| Partly Cloudy (1) | 336 | An animated short film depicting a relationship between a cloud that makes baby animals and the stork that delivers them. |
| Basketball Game (1) | 188 | A basketball team launches a 15-point comeback run to try to advance in a tournament. |
| Astronauts in Space (2) | 338 | Astronauts discuss their experience being in space. |
| Superhero Music Video (2) | 202 | A music video in which a girl pretends to be a superhero to escape bullying in her day-to-day life. |
| Office Music Video (3) | 319 | A music video in which a man deals with bullying at his workplace. |
| Bop-It (3) | 180 | A humorous skit in which kids play with a bop-it that asks them to cough and sneeze on the toy instead of the typical bop-it instructions. |

**Preprocessing Information**

As requested by the developers of fMRIPrep, the following boilerplate details the preprocessing performed in this manuscript.

Results included in this manuscript come from preprocessing performed using *fMRIPrep* 20.2.6 ((Esteban et al., 2019; Esteban, Markiewicz, Goncalves, et al., 2021) RRID:SCR_016216), which is based on *Nipype* 1.7.0 ((Esteban, Markiewicz, Burns, et al., 2021; Gorgolewski et al., 2011) RRID:SCR_002502).

**Anatomical data preprocessing**

A total of 1 T1-weighted (T1w) images were found within the input BIDS dataset. The T1-weighted (T1w) image was corrected for intensity non-uniformity (INU) with N4BiasFieldCorrection (Tustison et al., 2010), distributed with ANTs 2.3.3 ((Avants et al., 2008) RRID:SCR_004757), and used as T1w-reference throughout the workflow. The T1w-reference was then skull-stripped with a *Nipype* implementation of the antsBrainExtraction.sh workflow (from ANTs), using MNI152NLin2009cAsym as target template. Brain tissue segmentation of cerebrospinal fluid (CSF), white-matter (WM) and gray-matter (GM) was performed on the brain-extracted T1w using fast (FSL 5.0.9, RRID:SCR_002823, (Zhang et al., 2001)). Brain surfaces were reconstructed using recon-all (FreeSurfer 6.0.1, RRID:SCR_001847, (Dale et al., 1999)), and the brain mask estimated previously was refined with a custom variation of the method to reconcile ANTs-derived and FreeSurfer-derived segmentations of the cortical gray-matter of Mindboggle (RRID:SCR_002438, (Klein et al., 2017)). Volume-based spatial normalization to three standard spaces (MNIPediatricAsym:cohort-5, MNI152NLin6Asym, MNI152NLin2009cAsym) was performed through nonlinear registration with antsRegistration (ANTs 2.3.3), using brain-extracted versions of both T1w reference and the T1w template. The following templates were selected for spatial normalization: *MNI’s unbiased standard MRI template for pediatric data from the 4.5 to 18.5y age range* [RRID:SCR_008796; TemplateFlow ID: MNIPediatricAsym:cohort-5], *FSL’s MNI ICBM 152 non-linear 6th Generation Asymmetric Average Brain Stereotaxic Registration Model* [(Evans et al., 2012) RRID:SCR_002823; TemplateFlow ID: MNI152NLin6Asym], *ICBM 152 Nonlinear Asymmetrical template version 2009c* [(Fonov et al., 2009) RRID:SCR_008796; TemplateFlow ID: MNI152NLin2009cAsym],

**Functional data preprocessing**

For each of the 7 BOLD runs found per subject (across all tasks and sessions), the following preprocessing was performed. First, a reference volume and its skull-stripped version were generated using a custom methodology of *fMRIPrep*. A B0-nonuniformity map (or *fieldmap*) was estimated based on two (or more) echo-planar imaging (EPI) references with opposing phase-encoding directions, with 3dQwarp Cox and Hyde (1997) (AFNI 20160207). Based on the estimated susceptibility distortion, a corrected EPI (echo-planar imaging) reference was calculated for a more accurate co-registration with the anatomical reference. The BOLD reference was then co-registered to the T1w reference using bbregister (FreeSurfer) which implements boundary-based registration (Greve & Fischl, 2009). Co-registration was configured with six degrees of freedom. Head-motion parameters with respect to the BOLD reference (transformation matrices, and six corresponding rotation and translation parameters) are estimated before any spatiotemporal filtering using mcflirt (FSL 5.0.9, (Jenkinson et al., 2002)). BOLD runs were slice-time corrected to 0.559s (0.5 of slice acquisition range 0s-1.12s) using 3dTshift from AFNI 20160207 ((Cox & Hyde, 1997) RRID:SCR_005927). The BOLD time-series (including slice-timing correction when applied) were resampled onto their original, native space by applying a single, composite transform to correct for head-motion and susceptibility distortions. These resampled BOLD time-series will be referred to as *preprocessed BOLD in original space*, or just *preprocessed BOLD*. The BOLD time-series were resampled into several standard spaces, correspondingly generating the following *spatially-normalized, preprocessed BOLD runs*: MNIPediatricAsym:cohort-5, MNI152NLin6Asym. First, a reference volume and its skull-stripped version were generated using a custom methodology of *fMRIPrep*. Automatic removal of motion artifacts using independent component analysis (ICA-AROMA, (Pruim et al., 2015)) was performed on the *preprocessed BOLD on MNI space* time-series after removal of non-steady state volumes and spatial smoothing with an isotropic, Gaussian kernel of 6mm FWHM (full-width half-maximum). Corresponding “non-aggresively” denoised runs were produced after such smoothing. Additionally, the “aggressive” noise-regressors were collected and placed in the corresponding confounds file. Several confounding time-series were calculated based on the *preprocessed BOLD*: framewise displacement (FD), DVARS and three region-wise global signals. FD was computed using two formulations following Power (absolute sum of relative motions, (Power et al., 2014)) and Jenkinson (relative root mean square displacement between affines, (Jenkinson et al., 2002)). FD and DVARS are calculated for each functional run, both using their implementations in *Nipype* (following the definitions by Power et al. 2014). The three global signals are extracted within the CSF, the WM, and the whole-brain masks. Additionally, a set of physiological regressors were extracted to allow for component-based noise correction (*CompCor*,(Behzadi et al., 2007)). Principal components are estimated after high-pass filtering the *preprocessed BOLD* time-series (using a discrete cosine filter with 128s cut-off) for the two *CompCor* variants: temporal (tCompCor) and anatomical (aCompCor). tCompCor components are then calculated from the top 2% variable voxels within the brain mask. For aCompCor, three probabilistic masks (CSF, WM and combined CSF+WM) are generated in anatomical space. The implementation differs from that of Behzadi et al. in that instead of eroding the masks by 2 pixels on BOLD space, the aCompCor masks are subtracted a mask of pixels that likely contain a volume fraction of GM. This mask is obtained by dilating a GM mask extracted from the FreeSurfer’s *aseg* segmentation, and it ensures components are not extracted from voxels containing a minimal fraction of GM. Finally, these masks are resampled into BOLD space and binarized by thresholding at 0.99 (as in the original implementation). Components are also calculated separately within the WM and CSF masks. For each CompCor decomposition, the *k* components with the largest singular values are retained, such that the retained components’ time series are sufficient to explain 50 percent of variance across the nuisance mask (CSF, WM, combined, or temporal). The remaining components are dropped from consideration. The head-motion estimates calculated in the correction step were also placed within the corresponding confounds file. The confound time series derived from head motion estimates and global signals were expanded with the inclusion of temporal derivatives and quadratic terms for each (Satterthwaite et al., 2013). Frames that exceeded a threshold of 0.5 mm FD or 1.5 standardised DVARS were annotated as motion outliers. All resamplings can be performed with *a single interpolation step* by composing all the pertinent transformations (i.e. head-motion transform matrices, susceptibility distortion correction when available, and co-registrations to anatomical and output spaces). Gridded (volumetric) resamplings were performed using antsApplyTransforms (ANTs), configured with Lanczos interpolation to minimize the smoothing effects of other kernels (Lanczos, 1964). Non-gridded (surface) resamplings were performed using mri_vol2surf (FreeSurfer).

Many internal operations of *fMRIPrep* use *Nilearn* 0.6.2 ((Abraham et al., 2014), RRID:SCR_001362), mostly within the functional processing workflow. For more details of the pipeline, see the section corresponding to workflows in *fMRIPrep*’s documentation (https://fmriprep.readthedocs.io/en/latest/workflows.html).

**Supplemental EMA Information**

All participants received 5 prompts on weekends (10AM-12PM, 12-2PM, 2-5PM, 5-7PM, 7-9PM). Participants who were permitted to receive messages on a cellphone during the school day received 5 prompts on schooldays (7-11AM, 11AM-2PM, 2-5PM, 5-7PM, 7-9PM), while participants who were not permitted to receive messages on a cellphone during the school day received 4 prompts (6:45-9AM, 2-5PM, 5-7PM, 7-9PM). Out of the 92 participants in the present study, 39 participants received 5 prompts and 53 participants received 4 prompts. These prompts asked participants about their current mood and current or recent interactions. Only responses about the current or recent interaction were analyzed in this study.

When participants were enrolled in the EMA portion of the study, they were given a demonstration of an example survey completion by the experimenter. Participants were shown the survey prompts while the experimenter read each of the prompts out loud and clicked on example responses. Participants were also verbally given the following definition of what constituted a social interaction for the purposes of the surveys: “*A mutual interaction means there should be back and forth conversation between you and someone else, whether that be a friend, a classmate, a family member, a teacher, or anyone else you are with that day. The interaction needs to be more than 5 minutes, not a quick back and forth, like over text or quickly saying hello to someone in person. For example, maybe you have a conversation with your parent about how your school day went, or you’re working in a group with other classmates during the school day and you guys are speaking back and forth, or you are texting a friend and sending multiple texts back and forth. These are all* ***good*** *examples of a “mutual interaction” where you and whoever you are talking to are engaged in the conversation. However, maybe you are in class and your teacher is explaining a lesson and you are just listening and not contributing to a class conversation. This isn’t a back and forth interaction, so we wouldn’t want you to count that. Or, maybe you are scrolling through Instagram, but you are just looking at posts and not actually talking to anyone through the app. This also wouldn’t count as a “mutual interaction”. We also want to make sure you are thinking about people and not animals, so don’t consider any interactions between you and your pets. For these questions, you can consider in-person or online/virtual interactions, as long as they are mutual/have that “back and forth” conversation aspect to them.”* Following the demonstration and verbal instructions, youth were given the opportunity to ask questions.

As part of the EMA protocol, participants were asked if they were currently in an interaction or had been in one within the prompt window. If they responded yes, participants were asked to specify their interaction partner(s) by checking one or more of the following: close family member, teacher or coach, parent(s), close friend(s), other friend(s), someone I am dating, classmate(s) or teammate(s), sibling(s), other family member(s), or other. Interactions were designated as a ‘peer’ interaction if the participant selected that their interaction included a person or people in the following categories: close friend, other friend, classmate/teammate, someone they were dating, or ‘other’ response that could be coded as one of the above (e.g. bandmate). All other interactions were designated as ‘non-peer’ interactions.

Participants were then asked to respond how ‘close or connected’ they felt to the person or people with whom they were interacting, on a scale of 0 (not at all close) to 100 (extremely close). To measure interaction success, participants were then asked ‘How did you feel the interaction went?’ For the first four months of the study, this question was asked on a scale of 0 (very good) to 100 (very bad) but was later reversed to a scale of 0 (very bad) to 100 (very good). Each participant received their ten days of EMA prompts exclusively using the updated scale (0=very bad) or exclusively using the initial scale (0=very good, data for these participants was reverse scored after data collection to align with the rest of the sample). No participant received prompts with a mix of scales.

**Amendments to the Pre-Registered Analysis Plan.**

To model hypothesis 2 and 2a in line with approaches to modeling crossed random effects in intersubject correlation data (Chen et al., 2017), neural similarity was used as the outcome variable and mean interaction success was used as the predictor.

**Pre-Registered Hypothesis 1: Does Neural Similarity Predict Interaction Success?**

We conducted 12 multilevel models, one for each of the ROIs, as described in the methods (‘Neural Similarity-to-EMA Analysis’) and report the unstandardized coefficients for neural similarity in Supplemental Table S2.

*Supplemental Table S2. Neural Similarity Across All Videos Does Not Predict Interaction Success in a Set of Twelve Mentalizing- and Reward-Related ROIs.*

| ROI | *B*_neuralsim_ | St. Error | df | *t* | *p*_raw_ | *p*_corrected_ |
| --- | --- | --- | --- | --- | --- | --- |
| ACC | 107.2516 | 90.62816 | 85.11762 | 1.183425 | 0.239935 | 0.840882 |
| AMY | 105.5287 | 92.67187 | 83.76431 | 1.138734 | 0.25806 | 0.840882 |
| dmPFC | -19.3084 | 41.55022 | 84.52919 | -0.4647 | 0.643341 | 0.901549 |
| lATL | -2.5103 | 46.7435 | 85.2594 | -0.0537 | 0.957297 | 0.957297 |
| lOFC | -15.589 | 67.05319 | 85.70397 | -0.23249 | 0.816714 | 0.913159 |
| lTPJ | -22.2542 | 32.10113 | 84.85744 | -0.69325 | 0.490044 | 0.840882 |
| Precuneus | 12.06155 | 28.77878 | 87.42972 | 0.419112 | 0.676162 | 0.901549 |
| rATL | 8.886616 | 43.07656 | 83.08988 | 0.206298 | 0.837063 | 0.913159 |
| rTPJ | 22.02418 | 31.18418 | 85.819 | 0.706261 | 0.481939 | 0.840882 |
| rVFC | -76.5041 | 65.68917 | 82.54743 | -1.16464 | 0.24752 | 0.840882 |
| vmPFC | -62.06 | 74.93839 | 81.01107 | -0.82815 | 0.41002 | 0.840882 |
| VS | 132.7684 | 191.7424 | 86.81619 | 0.692431 | 0.490515 | 0.840882 |

Follow-up analyses were conducted using neural similarity calculated for each of the six videos (Supplemental Tables S3-S8).

*Supplemental Table S3. Neural Similarity in the Partly Cloudy Video Does Not Predict Interaction Success in a Set of Twelve Mentalizing- and Reward-Related ROIs.*

| ROI | *B*_neuralsim_ | St. Error | df | *t* | *p*_raw_ | *p*_corrected_ |
| --- | --- | --- | --- | --- | --- | --- |
| ACC | 86.12352 | 56.67592 | 79.85619 | 1.519579 | 0.132566 | 0.48236 |
| AMY | 60.76001 | 42.93756 | 83.00145 | 1.415078 | 0.160787 | 0.48236 |
| dmPFC | -12.7493 | 32.35272 | 82.71098 | -0.39407 | 0.694541 | 0.757681 |
| lATL | -5.72007 | 29.22361 | 83.57781 | -0.19573 | 0.845293 | 0.845293 |
| lOFC | -28.7096 | 40.61664 | 81.68389 | -0.70684 | 0.481676 | 0.757681 |
| lTPJ | -18.0187 | 16.87018 | 82.35001 | -1.06808 | 0.288606 | 0.636058 |
| Precuneus | 6.578935 | 15.11019 | 84.93661 | 0.435397 | 0.664379 | 0.757681 |
| rATL | -10.7282 | 26.93506 | 81.37421 | -0.3983 | 0.691452 | 0.757681 |
| rTPJ | -8.28944 | 17.5763 | 81.95364 | -0.47163 | 0.638447 | 0.757681 |
| rVFC | -33.8493 | 22.60982 | 81.77186 | -1.49711 | 0.138216 | 0.48236 |
| vmPFC | -37.8263 | 37.64867 | 80.87429 | -1.00472 | 0.318029 | 0.636058 |
| VS | 250.0266 | 112.1363 | 83.66603 | 2.229667 | 0.028448 | 0.341382 |

*Supplemental Table S4. Neural Similarity in the Basketball Game Video Does Not Predict Interaction Success in a Set of Twelve Mentalizing- and Reward-Related ROIs.*

| ROI | *B*_neuralsim_ | St. Error | df | *t* | *p*_raw_ | *p*_corrected_ |
| --- | --- | --- | --- | --- | --- | --- |
| ACC | 9.848298 | 63.08796 | 81.43942 | 0.156104 | 0.876337 | 0.876337 |
| AMY | -28.9445 | 78.27501 | 82.06397 | -0.36978 | 0.712499 | 0.777272 |
| dmPFC | 58.75032 | 54.24838 | 82.34533 | 1.082987 | 0.281975 | 0.483386 |
| lATL | 23.11174 | 40.28715 | 81.19983 | 0.573675 | 0.567773 | 0.757031 |
| lOFC | -38.7493 | 63.67684 | 84.82882 | -0.60853 | 0.544461 | 0.757031 |
| lTPJ | 57.52423 | 33.13331 | 81.85554 | 1.736145 | 0.086301 | 0.465184 |
| Precuneus | 34.7621 | 28.59536 | 81.61701 | 1.215655 | 0.227621 | 0.483386 |
| rATL | 52.60412 | 35.14956 | 80.43587 | 1.49658 | 0.138416 | 0.465184 |
| rTPJ | 67.78224 | 27.26268 | 80.53314 | 2.486265 | 0.014977 | 0.179729 |
| rVFC | 87.08147 | 75.58544 | 80.24252 | 1.152093 | 0.252704 | 0.483386 |
| vmPFC | 79.64655 | 55.48429 | 79.747 | 1.435479 | 0.155061 | 0.465184 |
| VS | 34.94705 | 89.34414 | 82.96956 | 0.391151 | 0.696688 | 0.777272 |

*Supplemental Table S5. Neural Similarity in the Astronauts in Space video Does Not Predict Interaction Success in a Set of Twelve Mentalizing- and Reward-Related ROIs.*

| ROI | *B*_neuralsim_ | St. Error | df | *t* | *p*_raw_ | *p*_corrected_ |
| --- | --- | --- | --- | --- | --- | --- |
| ACC | 50.0872 | 79.13988 | 77.41999 | 0.632895 | 0.528668 | 0.918395 |
| AMY | 54.47323 | 66.48295 | 77.7992 | 0.819356 | 0.415086 | 0.918395 |
| dmPFC | -50.0045 | 54.15374 | 76.84189 | -0.92338 | 0.358699 | 0.918395 |
| lATL | -4.28972 | 33.85346 | 76.46964 | -0.12671 | 0.899499 | 0.918395 |
| lOFC | -16.3503 | 92.48928 | 78.01073 | -0.17678 | 0.860139 | 0.918395 |
| lTPJ | 7.548196 | 35.59433 | 76.75741 | 0.212062 | 0.832621 | 0.918395 |
| Precuneus | 7.231444 | 42.7511 | 78.13325 | 0.169152 | 0.866114 | 0.918395 |
| rATL | 22.86575 | 34.29084 | 76.66864 | 0.666818 | 0.50689 | 0.918395 |
| rTPJ | 31.7309 | 37.48184 | 77.11353 | 0.846567 | 0.399856 | 0.918395 |
| rVFC | -143.052 | 58.61085 | 75.5308 | -2.44071 | 0.017001 | 0.204017 |
| vmPFC | -28.2228 | 66.0908 | 75.44065 | -0.42703 | 0.670574 | 0.918395 |
| VS | 13.92056 | 135.4397 | 79.68805 | 0.102781 | 0.918395 | 0.918395 |

*Supplemental Table S6. Neural Similarity in the Superhero Music Video Does Not Predict Interaction Success in a Set of Twelve Mentalizing- and Reward-Related ROIs.*

| ROI | *B*_neuralsim_ | St. Error | df | *t* | *p*_raw_ | *p*_corrected_ |
| --- | --- | --- | --- | --- | --- | --- |
| ACC | 7.690934 | 61.59845 | 76.83322 | 0.124856 | 0.900964 | 0.984349 |
| AMY | -4.76145 | 47.2866 | 77.41057 | -0.10069 | 0.920054 | 0.984349 |
| dmPFC | -22.645 | 22.8565 | 76.56302 | -0.99075 | 0.324929 | 0.984349 |
| lATL | -2.63349 | 45.55478 | 78.70777 | -0.05781 | 0.954047 | 0.984349 |
| lOFC | 8.670284 | 41.00088 | 77.02278 | 0.211466 | 0.833082 | 0.984349 |
| lTPJ | -18.8102 | 22.77298 | 76.85128 | -0.82599 | 0.411368 | 0.984349 |
| Precuneus | -0.36151 | 18.36981 | 78.17265 | -0.01968 | 0.984349 | 0.984349 |
| rATL | -5.86559 | 27.31203 | 76.91022 | -0.21476 | 0.830521 | 0.984349 |
| rTPJ | 15.48512 | 17.60795 | 78.77912 | 0.879439 | 0.381838 | 0.984349 |
| rVFC | -77.0449 | 54.62037 | 76.98137 | -1.41055 | 0.162405 | 0.974428 |
| vmPFC | -111.964 | 44.05759 | 77.75215 | -2.5413 | 0.013034 | 0.156409 |
| VS | -24.7965 | 92.87007 | 77.35122 | -0.267 | 0.790178 | 0.984349 |

*Supplemental Table S7. Neural Similarity in the Office Music Video Does Not Predict Interaction Success in a Set of Twelve Mentalizing- and Reward-Related ROIs.*

| ROI | *B*_neuralsim_ | St. Error | df | *t* | *p*_raw_ | *p*_corrected_ |
| --- | --- | --- | --- | --- | --- | --- |
| ACC | 66.27471 | 57.76848 | 79.51049 | 1.147247 | 0.25472 | 0.988075 |
| AMY | 61.93613 | 57.41969 | 79.25997 | 1.078657 | 0.284013 | 0.988075 |
| dmPFC | -6.3143 | 19.19734 | 79.24125 | -0.32892 | 0.743088 | 0.988075 |
| lATL | 4.329566 | 32.92928 | 81.23474 | 0.131481 | 0.89572 | 0.988075 |
| lOFC | 1.008265 | 33.44176 | 80.72358 | 0.03015 | 0.976022 | 0.988075 |
| lTPJ | -18.2684 | 19.3957 | 80.86456 | -0.94188 | 0.349059 | 0.988075 |
| Precuneus | -6.88272 | 19.78165 | 80.72258 | -0.34793 | 0.728795 | 0.988075 |
| rATL | 0.432879 | 28.87102 | 80.05009 | 0.014994 | 0.988075 | 0.988075 |
| rTPJ | -9.31524 | 21.87572 | 81.36792 | -0.42583 | 0.671359 | 0.988075 |
| rVFC | -2.28636 | 44.53701 | 80.42074 | -0.05134 | 0.959185 | 0.988075 |
| vmPFC | -69.036 | 48.56981 | 80.21996 | -1.42138 | 0.159083 | 0.988075 |
| VS | 16.47892 | 99.83424 | 81.30984 | 0.165063 | 0.869304 | 0.988075 |

*Supplemental Table S8. Neural Similarity in the Bop-It Video Does Not Predict Interaction Success in a Set of Twelve Mentalizing- and Reward-Related ROIs.*

| ROI | *B*_neuralsim_ | St. Error | df | *t* | *p*_raw_ | *p*_corrected_ |
| --- | --- | --- | --- | --- | --- | --- |
| ACC | 46.85037 | 32.71023 | 80.73801 | 1.432285 | 0.155923 | 0.916234 |
| AMY | -15.7078 | 61.03719 | 79.6895 | -0.25735 | 0.797573 | 0.916234 |
| dmPFC | 10.08914 | 20.56403 | 81.56682 | 0.490621 | 0.625012 | 0.916234 |
| lATL | 13.66841 | 20.92079 | 83.288 | 0.653341 | 0.515336 | 0.916234 |
| lOFC | 1.137094 | 45.95497 | 78.79458 | 0.024744 | 0.980322 | 0.980322 |
| lTPJ | 3.89554 | 19.21788 | 79.94879 | 0.202704 | 0.839881 | 0.916234 |
| Precuneus | 11.81464 | 16.19958 | 84.67128 | 0.729317 | 0.46782 | 0.916234 |
| rATL | 7.845638 | 20.15546 | 82.25508 | 0.389256 | 0.698093 | 0.916234 |
| rTPJ | 8.788966 | 19.02868 | 81.20959 | 0.46188 | 0.645402 | 0.916234 |
| rVFC | 12.60263 | 29.60667 | 79.47628 | 0.425669 | 0.6715 | 0.916234 |
| vmPFC | 34.2432 | 31.62381 | 79.21504 | 1.08283 | 0.282169 | 0.916234 |
| VS | -101.885 | 81.52941 | 79.18144 | -1.24967 | 0.215102 | 0.916234 |

**Pre-Registered Hypothesis 2: Does the Relation Between Neural Similarity and Interaction Success Significantly Follow an Anna Karenina Model?**

We tested whether the relations between neural similarity and interaction success more specifically followed an Anna Karenina model, such that individuals with better interaction success were all neurally alike, while individuals who reported worse interaction success were all neurally idiosyncratic (pre-registered hypothesis 2). None of the twelve ROIs had significant fit for this model. We report the standardized coefficients for interaction success in Supplemental Table S9.

*Supplemental Table S9. Interaction Success and Neural Similarity Across All Videos Do Not Significantly Follow an Anna Karenina Model in a Set of Twelve Mentalizing- and Reward-Related ROIs.*

| ROI | β_meansuccess_ | St. Error | df | *t* | *p*_raw_ | *p*_corrected_ |
| --- | --- | --- | --- | --- | --- | --- |
| ACC | 0.051605 | 0.048746 | 4185 | 1.058637 | 0.289826 | 0.75306 |
| AMY | 0.063457 | 0.05246 | 4185 | 1.209621 | 0.226493 | 0.75306 |
| dmPFC | -0.03486 | 0.071123 | 4185 | -0.49011 | 0.624084 | 0.852607 |
| lATL | -0.00293 | 0.074925 | 4185 | -0.03904 | 0.96886 | 0.96886 |
| lOFC | -0.02309 | 0.06306 | 4185 | -0.36621 | 0.71423 | 0.852607 |
| lTPJ | -0.0357 | 0.076911 | 4185 | -0.4642 | 0.64253 | 0.852607 |
| Precuneus | 0.036592 | 0.08342 | 4185 | 0.438642 | 0.660944 | 0.852607 |
| rATL | 0.02061 | 0.074321 | 4185 | 0.277309 | 0.781556 | 0.852607 |
| rTPJ | 0.050893 | 0.078491 | 4185 | 0.648401 | 0.516761 | 0.852607 |
| rVFC | -0.05452 | 0.054114 | 4185 | -1.00745 | 0.313775 | 0.75306 |
| vmPFC | -0.06618 | 0.054451 | 4185 | -1.2154 | 0.224283 | 0.75306 |
| VS | 0.037268 | 0.031527 | 4185 | 1.182102 | 0.237233 | 0.75306 |

**Pre-Registered Hypothesis 2a: Does the Relation Between Neural Similarity and Interaction Success Significantly Follow an Anna Karenina Model Above and Beyond Self-Reported Video Preference Similarity?**

We also tested whether the relations between neural similarity and interaction success held controlling for self-reported video preference similarity among participants (pre-registered hypothesis 2a). None of the twelve ROIs had significant fit for this model. We report the standardized coefficients for interaction success in Supplemental Table S10.

*Supplemental Table S10. Interaction Success and Neural Similarity Across All Videos Do Not Significantly Follow an Anna Karenina Model in a Set of Twelve Mentalizing- and Reward-Related ROIs, Accounting for Similarity in Self-Reported Video Enjoyment.*

| ROI | β_meansuccess_ | St. Error | df | *t* | *p*_raw_ | *p*_corrected_ |
| --- | --- | --- | --- | --- | --- | --- |
| ACC | 0.051643 | 0.048758 | 4184 | 1.059161 | 0.289588 | 0.76103 |
| AMY | 0.062954 | 0.05239 | 4184 | 1.201643 | 0.22957 | 0.76103 |
| dmPFC | -0.035 | 0.071063 | 4184 | -0.49253 | 0.62237 | 0.84487 |
| lATL | -0.00325 | 0.074819 | 4184 | -0.04346 | 0.965338 | 0.96534 |
| lOFC | -0.02411 | 0.063474 | 4184 | -0.37988 | 0.704057 | 0.84487 |
| lTPJ | -0.03634 | 0.077041 | 4184 | -0.47168 | 0.637177 | 0.84487 |
| Precuneus | 0.036275 | 0.083503 | 4184 | 0.434414 | 0.66401 | 0.84487 |
| rATL | 0.020295 | 0.074317 | 4184 | 0.273085 | 0.784801 | 0.85615 |
| rTPJ | 0.050616 | 0.078568 | 4184 | 0.644234 | 0.519459 | 0.84487 |
| rVFC | -0.05423 | 0.054201 | 4184 | -1.00056 | 0.317098 | 0.76103 |
| vmPFC | -0.06633 | 0.054441 | 4184 | -1.21838 | 0.22315 | 0.76103 |
| VS | 0.037285 | 0.031545 | 4184 | 1.18195 | 0.237293 | 0.76103 |

**Hypothesis 1a: Does Youth-Reported Closeness Interact with Neural Similarity to Predict Interaction Success?**

We conducted 12 multilevel models, one for each of the ROIs, as described in the methods (Neural Similarity-to-EMA Analysis) and report the unstandardized coefficients for the interaction between neural similarity and person-centered closeness in Supplemental Table S11. There were no significant interactions between neural similarity and person-mean closeness.

*Supplemental Table S11. Neural Similarity in rTPJ and lOFC Significantly Interacts with Person-Centered Closeness to Predict Interaction Success.*

| ROI | *B*_closeXnSim_ | St. Error | df | *t* | *p*_raw_ | *p*_corrected_ |
| --- | --- | --- | --- | --- | --- | --- |
| ACC | 1.48861 | 1.07803 | 2405.733 | 1.38086 | 0.16745 | 0.22327 |
| AMY | 0.39558 | 1.30286 | 2405.894 | 0.30363 | 0.76144 | 0.76144 |
| dmPFC | 0.8073 | 0.54922 | 2405.294 | 1.46991 | 0.14172 | 0.22327 |
| lATL | 0.91317 | 0.58434 | 2405.061 | 1.56274 | 0.11825 | 0.22327 |
| lOFC | 2.69178 | 0.90762 | 2405.399 | 2.96577 | 0.00305 | 0.01829 |
| lTPJ | 0.68921 | 0.41669 | 2405.062 | 1.65403 | 0.09825 | 0.22327 |
| Precuneus | -0.13572 | 0.35499 | 2405.518 | -0.38231 | 0.70226 | 0.76144 |
| rATL | 0.7452 | 0.53613 | 2406.031 | 1.38997 | 0.16467 | 0.22327 |
| rTPJ | 1.59161 | 0.40113 | 2405.209 | 3.96785 | 0.00007 | 0.0009 |
| rVFC | -0.40255 | 0.80376 | 2406.175 | -0.50083 | 0.61654 | 0.73985 |
| vmPFC | 1.6518 | 0.98558 | 2405.651 | 1.67596 | 0.09388 | 0.22327 |
| VS | 3.78231 | 2.58665 | 2405.163 | 1.46224 | 0.14381 | 0.22327 |

Follow-up analyses were conducted using neural similarity calculated for each of the six videos (Supplemental Tables S12-S17).

*Supplemental Table S12. Interactions Between Closeness and Neural Similarity in the Partly Cloudy Video in a Set of Twelve Mentalizing- and Reward-Related ROIs.*

| ROI | *B*_closeXnSim_ | St. Error | df | *t* | *p*_raw_ | *p*_corrected_ |
| --- | --- | --- | --- | --- | --- | --- |
| ACC | 0.7387 | 0.74349 | 2358.605 | 0.99358 | 0.320529534 | 0.384635 |
| AMY | 0.0036 | 0.60027 | 2359.84 | 0.00598 | 0.995229466 | 0.995229 |
| dmPFC | 0.5210 | 0.44844 | 2359.08 | 1.16171 | 0.245470895 | 0.327295 |
| lATL | 0.9083 | 0.40226 | 2358.11 | 2.257987 | 0.024037564 | 0.072113 |
| lOFC | 1.5998 | 0.52915 | 2358.65 | 3.02341 | 0.002526365 | 0.011904 |
| lTPJ | 0.6886 | 0.22421 | 2358.49 | 3.07112 | 0.002156903 | 0.011904 |
| Precuneus | 0.0581 | 0.18164 | 2358.84 | 0.31961 | 0.74929252 | 0.81741 |
| rATL | 0.6184 | 0.34610 | 2358.16 | 1.784656 | 0.074445712 | 0.148891 |
| rTPJ | 0.6463 | 0.21738 | 2358.22 | 2.97331 | 0.002975894 | 0.011904 |
| rVFC | -0.5001 | 0.29660 | 2359.18 | -1.68622 | 0.09188566 | 0.157518 |
| vmPFC | 1.0987 | 0.53642 | 2357.57 | 2.048204 | 0.04065051 | 0.097561 |
| VS | 1.8702 | 1.48632 | 2358.13 | 1.258265 | 0.208420649 | 0.312631 |

*Supplemental Table S13. Interactions Between Closeness and Neural Similarity in the Basketball Video in a Set of Twelve Mentalizing- and Reward-Related ROIs.*

| ROI | *B*_closeXnSim_ | St. Error | df | *t* | *p*_raw_ | *p*_corrected_ |
| --- | --- | --- | --- | --- | --- | --- |
| ACC | 1.25899 | 0.76024 | 2358.955 | 1.65604 | 0.09785 | 0.29354 |
| AMY | -1.04674 | 1.00149 | 2359.215 | -1.04519 | 0.29604 | 0.50750 |
| dmPFC | 1.99198 | 0.65371 | 2359.311 | 3.04721 | 0.00234 | 0.01085 |
| lATL | -0.82826 | 0.53886 | 2359.005 | -1.53705 | 0.12442 | 0.29860 |
| lOFC | -0.45462 | 0.83743 | 2358.39 | -0.54288 | 0.58726 | 0.70472 |
| lTPJ | 0.38104 | 0.42741 | 2357.195 | 0.89152 | 0.37274 | 0.55911 |
| Precuneus | -1.01259 | 0.33735 | 2357.73 | -3.00162 | 0.00271 | 0.01085 |
| rATL | -0.52985 | 0.42919 | 2356.615 | -1.23455 | 0.21712 | 0.43424 |
| rTPJ | 0.20377 | 0.35583 | 2358.083 | 0.57268 | 0.56691 | 0.70472 |
| rVFC | 0.08690 | 0.95193 | 2358.648 | 0.09129 | 0.92727 | 0.94974 |
| vmPFC | 0.04406 | 0.69898 | 2358.299 | 0.06304 | 0.94974 | 0.94974 |
| VS | 4.39063 | 1.20817 | 2358.551 | 3.63413 | 0.00028 | 0.00342 |

*Supplemental Table S14. Interactions Between Closeness and Neural Similarity in the Astronauts in Space Video in a Set of Twelve Mentalizing- and Reward-Related ROIs.*

| ROI | *B*_closeXnSim_ | St. Error | df | *t* | *p*_raw_ | *p*_corrected_ |
| --- | --- | --- | --- | --- | --- | --- |
| ACC | -0.95207 | 0.92402 | 2313.09 | -1.03035 | 0.30295 | 0.45443 |
| AMY | 0.49125 | 0.91932 | 2313.26 | 0.53436 | 0.59315 | 0.72693 |
| dmPFC | 0.27525 | 0.63832 | 2313.40 | 0.43121 | 0.66635 | 0.72693 |
| lATL | 0.69546 | 0.37438 | 2313.09 | 1.85763 | 0.06335 | 0.12670 |
| lOFC | 4.68900 | 1.13607 | 2313.18 | 4.12739 | 0.00004 | 0.00046 |
| lTPJ | 0.83536 | 0.43941 | 2313.23 | 1.90109 | 0.05741 | 0.12670 |
| Precuneus | 0.00389 | 0.56190 | 2312.46 | 0.00693 | 0.99448 | 0.99448 |
| rATL | 0.20274 | 0.39703 | 2314.22 | 0.51064 | 0.60965 | 0.72693 |
| rTPJ | 1.51827 | 0.48092 | 2313.02 | 3.15701 | 0.00161 | 0.00646 |
| rVFC | 1.87053 | 0.76324 | 2312.95 | 2.45077 | 0.01433 | 0.04299 |
| vmPFC | 0.87311 | 0.74919 | 2313.49 | 1.16540 | 0.24398 | 0.41825 |
| VS | -5.8262 | 1.71304 | 2313.07 | -3.4011 | 0.00068 | 0.00410 |

*Supplemental Table S15. Interactions Between Closeness and Neural Similarity in the Superhero Music Video in a Set of Twelve Mentalizing- and Reward-Related ROIs.*

| ROI | *B*_closeXnSim_ | St. Error | df | *t* | *p*_raw_ | *p*_corrected_ |
| --- | --- | --- | --- | --- | --- | --- |
| ACC | 0.51970 | 0.66340 | 2313.06 | 0.78340 | 0.43347 | 0.70998 |
| AMY | -0.50798 | 0.62363 | 2313.18 | -0.81455 | 0.41541 | 0.70998 |
| dmPFC | 0.00922 | 0.30160 | 2313.24 | 0.03058 | 0.97561 | 0.97561 |
| lATL | -0.66061 | 0.51675 | 2312.90 | -1.27840 | 0.20124 | 0.60371 |
| lOFC | 1.89473 | 0.54510 | 2312.92 | 3.47595 | 0.00052 | 0.00622 |
| lTPJ | -0.8149 | 0.25473 | 2313.39 | -3.1990 | 0.00140 | 0.00839 |
| Precuneus | -0.33310 | 0.21875 | 2312.73 | -1.52279 | 0.12795 | 0.51179 |
| rATL | 0.20960 | 0.33573 | 2312.84 | 0.62431 | 0.53249 | 0.70998 |
| rTPJ | 0.19684 | 0.22180 | 2312.47 | 0.88749 | 0.37491 | 0.70998 |
| rVFC | -0.41908 | 0.65488 | 2312.87 | -0.63994 | 0.52228 | 0.70998 |
| vmPFC | 0.18427 | 0.55672 | 2313.09 | 0.33100 | 0.74068 | 0.88881 |
| VS | -0.22992 | 1.12929 | 2313.42 | -0.20360 | 0.83869 | 0.91493 |

*Supplemental Table S16. Interactions Between Closeness and Neural Similarity in the Office Music Video in a Set of Twelve Mentalizing- and Reward-Related ROIs.*

| ROI | *B*_closeXnSim_ | St. Error | df | *t* | *p*_raw_ | *p*_corrected_ |
| --- | --- | --- | --- | --- | --- | --- |
| ACC | 0.83234 | 0.73960 | 2359.18 | 1.12539 | 0.260538 | 0.43786 |
| AMY | 0.80371 | 0.73229 | 2358.83 | 1.09754 | 0.272516 | 0.43786 |
| dmPFC | 0.06673 | 0.25654 | 2358.74 | 0.26013 | 0.794790 | 0.79479 |
| lATL | 0.45445 | 0.43110 | 2359.15 | 1.05418 | 0.291910 | 0.43786 |
| lOFC | 0.68204 | 0.41775 | 2358.40 | 1.63265 | 0.102676 | 0.43786 |
| lTPJ | -0.07017 | 0.25585 | 2358.98 | -0.27425 | 0.783916 | 0.79479 |
| Precuneus | -0.40726 | 0.25989 | 2359.88 | -1.56705 | 0.117237 | 0.43786 |
| rATL | 0.51272 | 0.37759 | 2359.46 | 1.35787 | 0.174635 | 0.43786 |
| rTPJ | 1.30497 | 0.28308 | 2359.08 | 4.60984 | 0.000004 | 0.00005 |
| rVFC | -0.40192 | 0.59361 | 2359.27 | -0.67709 | 0.498418 | 0.59810 |
| vmPFC | 0.78430 | 0.66382 | 2359.51 | 1.18150 | 0.237522 | 0.43786 |
| VS | 1.22527 | 1.56615 | 2359.55 | 0.78235 | 0.434090 | 0.57879 |

*Supplemental Table S17. Interactions Between Closeness and Neural Similarity in the Bop-It Video in a Set of Twelve Mentalizing- and Reward-Related ROIs.*

| ROI | *B*_closeXnSim_ | St. Error | df | *t* | *p*_raw_ | *p*_corrected_ |
| --- | --- | --- | --- | --- | --- | --- |
| ACC | 1.32446 | 0.48703 | 2359.59 | 2.71945 | 0.00659 | 0.05381 |
| AMY | -2.15196 | 0.82280 | 2359.19 | -2.61541 | 0.00897 | 0.05381 |
| dmPFC | 0.05889 | 0.30117 | 2359.00 | 0.19553 | 0.84499 | 0.84499 |
| lATL | 0.43585 | 0.27704 | 2359.32 | 1.57323 | 0.11580 | 0.23160 |
| lOFC | -1.31083 | 0.59753 | 2359.75 | -2.19376 | 0.02835 | 0.11340 |
| lTPJ | 0.32610 | 0.25653 | 2358.54 | 1.27121 | 0.20378 | 0.34934 |
| Precuneus | -0.15263 | 0.21533 | 2360.02 | -0.70882 | 0.47850 | 0.63801 |
| rATL | 0.11356 | 0.28150 | 2360.71 | 0.40341 | 0.68669 | 0.74911 |
| rTPJ | 0.25666 | 0.23025 | 2360.11 | 1.11469 | 0.26510 | 0.39765 |
| rVFC | -0.20510 | 0.35965 | 2361.43 | -0.57030 | 0.56853 | 0.68224 |
| vmPFC | 0.60881 | 0.38491 | 2359.75 | 1.58170 | 0.11385 | 0.23160 |
| VS | 2.05284 | 1.03347 | 2358.83 | 1.98635 | 0.04711 | 0.14133 |

**Exploratory Analysis: Does Whether or Not an Interaction is with a Peer Interact with Neural Similarity to Predict Interaction Success?**

We conducted 12 multilevel models to predict interaction success, one for each of the ROIs, as described in the methods (Neural Similarity-to-EMA Analysis) and report the unstandardized coefficients for the interaction between neural similarity and person-centered social partner (peer/non-peer) in Supplemental Table S18. There were no significant interactions between neural similarity and person-mean peer interaction.

*Supplemental Table S18. Interactions Between Peer Interaction and Neural Similarity Across All Videos in a Set of Twelve Mentalizing- and Reward-Related ROIs.*

| ROI | *B*_peerXnSim_ | St. Error | df | *t* | *p*_raw_ | *p*_corrected_ |
| --- | --- | --- | --- | --- | --- | --- |
| ACC | 68.76124 | 73.98184 | 74.22303 | 0.929434 | 0.355677 | 0.388011 |
| AMY | -121.853 | 78.65188 | 69.54333 | -1.54927 | 0.125856 | 0.251712 |
| dmPFC | 46.61887 | 36.82627 | 79.2033 | 1.265914 | 0.209255 | 0.279007 |
| lATL | 78.03484 | 39.42419 | 75.5275 | 1.979365 | 0.05142 | 0.205678 |
| lOFC | 24.07908 | 59.26021 | 81.96633 | 0.406328 | 0.685561 | 0.685561 |
| lTPJ | 74.00673 | 26.83073 | 71.04303 | 2.758283 | 0.007383 | 0.088592 |
| Precuneus | 31.77075 | 25.06518 | 79.01663 | 1.267525 | 0.20869 | 0.279007 |
| rATL | 42.39559 | 39.48205 | 74.91249 | 1.073794 | 0.286362 | 0.343634 |
| rTPJ | 49.42538 | 27.15884 | 77.81832 | 1.819864 | 0.072625 | 0.217876 |
| rVFC | 88.05256 | 54.60439 | 72.49104 | 1.612555 | 0.111187 | 0.251712 |
| vmPFC | 84.84815 | 62.50555 | 68.22917 | 1.35745 | 0.179111 | 0.279007 |
| VS | -397.918 | 163.8053 | 74.14662 | -2.42921 | 0.017554 | 0.105325 |

We performed follow-up analyses using neural similarity calculated with each of the six videos (Supplemental Tables S19-S24).

*Supplemental Table S19. Interactions Between Peer Interaction and Neural Similarity in the Partly Cloudy Video in a Set of Twelve Mentalizing- and Reward-Related ROIs.*

| ROI | *B*_peerXnSim_ | St. Error | df | *t* | *p*_raw_ | *p*_corrected_ |
| --- | --- | --- | --- | --- | --- | --- |
| ACC | 55.31031 | 48.70399 | 72.29731 | 1.135642 | 0.259857 | 0.311828 |
| AMY | -71.0507 | 37.22115 | 68.64443 | -1.90888 | 0.060459 | 0.120918 |
| dmPFC | 58.11371 | 27.42102 | 71.03551 | 2.119312 | 0.037561 | 0.090145 |
| lATL | 58.68624 | 26.7323 | 77.05261 | 2.195331 | 0.03115 | 0.090145 |
| lOFC | -1.39359 | 35.49236 | 70.3237 | -0.03926 | 0.968791 | 0.968791 |
| lTPJ | 40.48259 | 14.93278 | 72.80801 | 2.710988 | 0.008362 | 0.048101 |
| Precuneus | 21.68453 | 13.09835 | 70.69618 | 1.655516 | 0.102253 | 0.153379 |
| rATL | 39.99517 | 22.46045 | 69.48595 | 1.780693 | 0.079334 | 0.136001 |
| rTPJ | 41.71682 | 15.15066 | 70.10562 | 2.753466 | 0.007504 | 0.048101 |
| rVFC | 49.8608 | 19.34164 | 70.71114 | 2.577899 | 0.012025 | 0.048101 |
| vmPFC | 36.50608 | 32.03795 | 73.49369 | 1.139464 | 0.25821 | 0.311828 |
| VS | -27.4694 | 99.92552 | 67.51259 | -0.2749 | 0.784234 | 0.855527 |

*Supplemental Table S20. Interactions Between Peer Interaction and Neural Similarity in the Basketball Video in a Set of Twelve Mentalizing- and Reward-Related ROIs.*

| ROI | *B*_peerXnSim_ | St. Error | df | *t* | *p*_raw_ | *p*_corrected_ |
| --- | --- | --- | --- | --- | --- | --- |
| ACC | -58.159 | 52.83635 | 70.8894 | -1.10074 | 0.274734 | 0.934094 |
| AMY | -52.2858 | 67.34289 | 68.87818 | -0.77641 | 0.440164 | 0.934094 |
| dmPFC | -22.3073 | 48.23438 | 69.08294 | -0.46248 | 0.645193 | 0.934094 |
| lATL | 28.10365 | 34.09539 | 69.61899 | 0.824265 | 0.412604 | 0.934094 |
| lOFC | 22.44285 | 55.61514 | 73.82046 | 0.403539 | 0.687719 | 0.934094 |
| lTPJ | 10.30643 | 29.16177 | 75.16406 | 0.353423 | 0.724761 | 0.934094 |
| Precuneus | -7.52777 | 23.46928 | 72.67426 | -0.32075 | 0.74932 | 0.934094 |
| rATL | 0.328403 | 31.08242 | 64.07848 | 0.010566 | 0.991603 | 0.991603 |
| rTPJ | -9.19423 | 24.1854 | 68.29413 | -0.38016 | 0.705009 | 0.934094 |
| rVFC | -18.0341 | 63.8382 | 68.77417 | -0.2825 | 0.778411 | 0.934094 |
| vmPFC | 3.529949 | 45.27591 | 65.39342 | 0.077965 | 0.938094 | 0.991603 |
| VS | -87.359 | 79.03579 | 69.2475 | -1.10531 | 0.272851 | 0.934094 |

*Supplemental Table S21. Interactions Between Peer Interaction and Neural Similarity in the Astronauts in Space Video in a Set of Twelve Mentalizing- and Reward-Related ROIs.*

| ROI | *B*_peerXnSim_ | St. Error | df | *t* | *p*_raw_ | *p*_corrected_ |
| --- | --- | --- | --- | --- | --- | --- |
| ACC | 1.522277 | 63.6314 | 66.00259 | 0.023923 | 0.980986 | 0.980986 |
| AMY | -32.337 | 57.29733 | 72.19172 | -0.56437 | 0.574251 | 0.980986 |
| dmPFC | -16.5928 | 47.01046 | 75.25746 | -0.35296 | 0.725105 | 0.980986 |
| lATL | -2.85616 | 26.54077 | 73.72217 | -0.10761 | 0.914594 | 0.980986 |
| lOFC | 48.8192 | 79.54554 | 79.21586 | 0.613726 | 0.541155 | 0.980986 |
| lTPJ | 11.11092 | 29.0827 | 73.27396 | 0.382046 | 0.703533 | 0.980986 |
| Precuneus | -11.3848 | 38.6988 | 75.83387 | -0.29419 | 0.769417 | 0.980986 |
| rATL | -17.026 | 30.53729 | 70.32925 | -0.55755 | 0.578924 | 0.980986 |
| rTPJ | -28.5298 | 32.71516 | 80.95139 | -0.87207 | 0.385752 | 0.980986 |
| rVFC | -38.9593 | 49.90219 | 76.06329 | -0.78071 | 0.437394 | 0.980986 |
| vmPFC | -11.5325 | 55.22662 | 69.69764 | -0.20882 | 0.835197 | 0.980986 |
| VS | -147.829 | 118.6141 | 72.21805 | -1.24631 | 0.21668 | 0.980986 |

*Supplemental Table S22. Interactions Between Peer Interaction and Neural Similarity in the Superhero Music Video in a Set of Twelve Mentalizing- and Reward-Related ROIs.*

| ROI | *B*_peerXnSim_ | St. Error | df | *t* | *p*_raw_ | *p*_corrected_ |
| --- | --- | --- | --- | --- | --- | --- |
| ACC | 10.7311 | 52.79897 | 70.36558 | 0.203244 | 0.839531 | 0.957682 |
| AMY | -63.6422 | 39.66628 | 72.62064 | -1.60444 | 0.112958 | 0.442395 |
| dmPFC | 1.693782 | 19.15112 | 69.29946 | 0.088443 | 0.92978 | 0.957682 |
| lATL | 85.44493 | 37.85 | 74.30065 | 2.257462 | 0.026917 | 0.32301 |
| lOFC | -27.6271 | 34.85273 | 67.78813 | -0.79268 | 0.430729 | 0.738393 |
| lTPJ | 3.80844 | 19.75487 | 72.28927 | 0.192785 | 0.847668 | 0.957682 |
| Precuneus | 0.821633 | 15.43426 | 77.39153 | 0.053234 | 0.957682 | 0.957682 |
| rATL | 20.51773 | 24.28166 | 73.45064 | 0.844989 | 0.400861 | 0.738393 |
| rTPJ | 5.082974 | 15.24946 | 75.83335 | 0.333322 | 0.739811 | 0.957682 |
| rVFC | 68.1579 | 45.49813 | 73.42922 | 1.498037 | 0.13841 | 0.442395 |
| vmPFC | 38.76543 | 37.98022 | 66.55838 | 1.020674 | 0.311105 | 0.738393 |
| VS | -118.719 | 81.09028 | 73.18529 | -1.46404 | 0.147465 | 0.442395 |

*Supplemental Table S23. Interactions Between Peer Interaction and Neural Similarity in the Office Music Video in a Set of Twelve Mentalizing- and Reward-Related ROIs.*

| ROI | *B*_peerXnSim_ | St. Error | df | *t* | *p*_raw_ | *p*_corrected_ |
| --- | --- | --- | --- | --- | --- | --- |
| ACC | 32.82917 | 46.78852 | 71.22389 | 0.70165 | 0.485184 | 0.988487 |
| AMY | -57.7836 | 44.7665 | 63.70589 | -1.29078 | 0.201446 | 0.67009 |
| dmPFC | 2.961903 | 16.25619 | 69.36199 | 0.182202 | 0.855956 | 0.988487 |
| lATL | 8.113371 | 28.55149 | 68.25489 | 0.284166 | 0.777143 | 0.988487 |
| lOFC | 17.01505 | 28.68039 | 79.89027 | 0.593264 | 0.55468 | 0.988487 |
| lTPJ | 23.09141 | 16.31497 | 66.07948 | 1.415351 | 0.161661 | 0.67009 |
| Precuneus | -9.41677 | 17.11833 | 74.09646 | -0.5501 | 0.583906 | 0.988487 |
| rATL | 2.53861 | 24.28728 | 70.5483 | 0.104524 | 0.91705 | 0.988487 |
| rTPJ | 22.9179 | 18.66423 | 74.20703 | 1.227905 | 0.223363 | 0.67009 |
| rVFC | 0.523436 | 36.13933 | 66.48793 | 0.014484 | 0.988487 | 0.988487 |
| vmPFC | 13.5749 | 40.1072 | 63.93114 | 0.338465 | 0.736122 | 0.988487 |
| VS | -171.098 | 84.72828 | 73.00412 | -2.01937 | 0.047121 | 0.565448 |

*Supplemental Table S24. Interactions Between Peer Interaction and Neural Similarity in the Bop-It Video in a Set of Twelve Mentalizing- and Reward-Related ROIs.*

| ROI | *B*_peerXnSim_ | St. Error | df | *t* | *p*_raw_ | *p*_corrected_ |
| --- | --- | --- | --- | --- | --- | --- |
| ACC | 14.51288 | 28.38087 | 71.7871 | 0.511362 | 0.610667 | 0.666182 |
| AMY | 30.93456 | 51.24198 | 73.18138 | 0.603696 | 0.547913 | 0.657769 |
| dmPFC | 34.84418 | 17.40524 | 79.91993 | 2.001936 | 0.048684 | 0.255398 |
| lATL | 27.40322 | 16.74406 | 66.84107 | 1.636594 | 0.106416 | 0.255398 |
| lOFC | -22.9501 | 38.02241 | 67.34002 | -0.60359 | 0.548141 | 0.657769 |
| lTPJ | 44.64007 | 15.40507 | 65.44524 | 2.897752 | 0.005108 | 0.061296 |
| Precuneus | 23.77647 | 13.93016 | 76.08818 | 1.706834 | 0.091931 | 0.255398 |
| rATL | 17.15492 | 18.18486 | 77.69252 | 0.943363 | 0.34842 | 0.522629 |
| rTPJ | 26.97015 | 15.5822 | 70.23097 | 1.730831 | 0.087873 | 0.255398 |
| rVFC | 29.31288 | 24.50221 | 68.07127 | 1.196336 | 0.235716 | 0.404084 |
| vmPFC | 33.2313 | 25.36049 | 59.90866 | 1.310357 | 0.195077 | 0.390154 |
| VS | -19.4841 | 69.62333 | 67.98888 | -0.27985 | 0.780442 | 0.780442 |

**Exploratory Analysis: Does Binarized Closeness Interact with Neural Similarity to Predict Interaction Success?**

We also evaluated interaction closeness as a categorical variable as follows.

Interactions were designated as a ‘close’ interaction if the participant selected that their interaction included a person or people in the following categories: ‘close friend’ or ‘close family member’. All other interactions were designated as ‘not close’ interactions. This variable was used as a moderator to conduct an exploratory extension of hypothesis 1a.

A multilevel model was constructed treating EMA observations as nested within individuals, and intercepts were modeled as randomly varying across individuals. Closeness was incorporated as person-mean and person-mean-centered predictors to distinguish between relations at the within and between person levels (Enders & Tofighi, 2007), and two interaction terms were modeled, one between the person-mean-centered closeness variable and neural similarity, and the other between the person-mean closeness variable and neural similarity. Age, gender, and group were included as covariates. Again, twelve models were evaluated, one for each of the twelve ROIs, and FDR correction was performed across the interaction coefficients of interest for the twelve models to account for multiple comparisons.

There were no ROIs in which neural similarity significantly interacted with the binarized closeness predictor to predict interaction success.

**Exploratory Analysis: Whole-Brain Parcellation**

Hypotheses were also tested using a 268 parcel whole-brain parcellation (Shen et al., 2013). First, calculation of TSNR was performed in AFNI using 3dtstat to identify parcels with low signal-to-noise ratio (Cox, 1996; Cox & Hyde, 1997). This analysis identified six parcels with low signal-to-noise, which were removed from further analysis. Models were constructed for each of the hypotheses and tested in each parcel separately, then corrected for multiple comparison across the 262 parcels used for analysis. For hypothesis 1, 2, and 2a, there were no parcels in which the relations survived correction for multiple comparisons.

For hypothesis 1a, testing whether youth-reported closeness interacted with neural similarity to predict interaction success, 40 parcels survived correction for multiple comparisons. Results overlapped with the ROI analysis for this hypothesis in the main text (Figure 3); parcels overlapping with the rTPJ and lOFC ROIs were identified. Additional regions including parcels overlapping with the insula and cerebellum emerged in same direction as the results presented in the main text, such that the relation between closeness and interaction success was stronger for individuals with higher neural similarity in those parcels. Some parcels, including a parcel overlapping with the amygdala, emerged in the opposite direction to the results presented in the main text, such that the relation between closeness and interaction success was weaker for individuals with higher neural similarity in those parcels. The full list of significant parcels is provided below in Supplemental Table S25.

*Supplemental Table S25. Interactions Between Closeness and Neural Similarity in a Whole-Brain Parcellation*

| Region (Hemisphere) - Parcel Number | *B*_closeXnSim_ | St. Error | df | *t* | *p*_raw_ | *p*_corrected_ |
| --- | --- | --- | --- | --- | --- | --- |
| Amygdala (R) - 99 | -5.24 | 1.62 | 2404.9 | -3.23 | 0.001 | 0.018 |
| Angular Gyrus (R) - 50 | 1.16 | 0.32 | 2405.3 | 3.58 | <0.001 | 0.007 |
| Brainstem (L) – 267 | 8.8 | 3.13 | 2405.4 | 2.81 | 0.005 | 0.037 |
| Brainstem (R) - 131 | 15.23 | 4.3 | 2405.4 | 3.54 | <0.001 | 0.008 |
| Caudate (L) - 257 | 5.45 | 1.93 | 2404.7 | 2.83 | 0.005 | 0.037 |
| Cerebellum (L) - 236 | 2.33 | 0.78 | 2406.3 | 2.99 | 0.003 | 0.028 |
| Cerebellum (L) - 238 | 3.04 | 0.79 | 2406.6 | 3.85 | <0.001 | 0.003 |
| Cerebellum (L) - 240 | -2.21 | 0.71 | 2405.5 | -3.1 | 0.002 | 0.022 |
| Cerebellum (L) - 241 | 3.33 | 1.11 | 2407.2 | 3 | 0.003 | 0.028 |
| Cerebellum (L) - 246 | 4.43 | 1.05 | 2405.5 | 4.22 | <0.001 | 0.002 |
| Cerebellum (L) - 255 | 8.7 | 2.19 | 2406.3 | 3.97 | <0.001 | 0.003 |
| Cerebellum (R) - 100 | 2.7 | 0.86 | 2405.4 | 3.14 | 0.002 | 0.021 |
| Cerebellum (R) - 107 | 4.67 | 1.61 | 2405.5 | 2.9 | 0.004 | 0.033 |
| Cerebellum (R) - 113 | 3.13 | 1.1 | 2406.1 | 2.85 | 0.004 | 0.035 |
| Cerebellum (R) - 114 | 2.56 | 0.92 | 2405.6 | 2.79 | 0.005 | 0.038 |
| Cerebellum (R) - 115 | 7.08 | 1.72 | 2406.1 | 4.11 | <0.001 | 0.002 |
| Cerebellum (R) - 116 | 5.3 | 1.54 | 2405.8 | 3.45 | 0.001 | 0.01 |
| Cerebellum (R) - 117 | 3.41 | 0.82 | 2405.2 | 4.16 | <0.001 | 0.002 |
| Frontal Operculum Cortex (R) - 20 | 2.23 | 0.73 | 2405.6 | 3.04 | 0.002 | 0.026 |
| Frontal Orbital Cortex (L) - 135 | 8.45 | 3.16 | 2405.6 | 2.67 | 0.008 | 0.049 |
| Frontal Pole (L) - 139 | 11.57 | 1.8 | 2405 | 6.43 | <0.001 | <0.001 |
| Frontal Pole (L) - 142 | 4.26 | 0.9 | 2405.1 | 4.7 | <0.001 | <0.001 |
| Frontal Pole (R) - 7 | 2.29 | 0.82 | 2406 | 2.81 | 0.005 | 0.037 |
| Insular Cortex (R) - 35 | 1.92 | 0.71 | 2405.3 | 2.71 | 0.007 | 0.045 |
| Insular Cortex (R) - 37 | 2.97 | 1.03 | 2405.7 | 2.9 | 0.004 | 0.033 |
| Lateral Occipital Cortex (L) - 209 | -0.67 | 0.24 | 2405.6 | -2.75 | 0.006 | 0.042 |
| Middle Temporal Gyrus (L) - 193 | 4.35 | 1.12 | 2404 | 3.88 | <0.001 | 0.003 |
| Paracingulate Gyrus (R) - 28 | 2.98 | 0.93 | 2405.5 | 3.2 | 0.001 | 0.019 |
| Parahippocampal Gyrus (L) - 234 | -4.86 | 1.62 | 2405.1 | -3 | 0.003 | 0.028 |
| Postcentral Gyrus (L) - 172 | 3.16 | 0.92 | 2403.9 | 3.42 | 0.001 | 0.01 |
| Postcentral Gyrus (R) - 39 | 3.19 | 0.99 | 2405.2 | 3.24 | 0.001 | 0.018 |
| Posterior Cingulate (L) - 227 | 2.31 | 0.64 | 2405.5 | 3.58 | <0.001 | 0.007 |
| Precentral Gyrus (L) - 159 | -6.97 | 1.95 | 2406 | -3.58 | <0.001 | 0.007 |
| Precentral Gyrus (L) - 167 | 3.75 | 1.3 | 2405.5 | 2.88 | 0.004 | 0.033 |
| Putamen (R) - 124 | 5.39 | 1.84 | 2406.1 | 2.92 | 0.003 | 0.033 |
| Subcallosal Cortex (L) - 136 | 9.84 | 2.54 | 2405.2 | 3.87 | <0.001 | 0.003 |
| Superior Frontal Gyrus (L) - 148 | 1.9 | 0.6 | 2405.4 | 3.16 | 0.002 | 0.021 |
| Thalamus (L) - 263 | 4.93 | 1.58 | 2405.7 | 3.12 | 0.002 | 0.021 |
| Thalamus (L) - 264 | 7.71 | 1.88 | 2405.9 | 4.09 | <0.001 | 0.002 |
| Thalamus (R) - 128 | 3.65 | 1.28 | 2406.3 | 2.86 | 0.004 | 0.035 |

**Exploratory Analysis: Within-Group Analyses – Non-Autistic Group**

Hypotheses were also tested within just the non-autistic group (*n*=67), using neural similarity values calculated from each non-autistic participant to every other non-autistic participant. For hypothesis 1, there were no regions across the twelve mentalizing/reward ROIs in which neural similarity significantly predicted interaction success in just the non-autistic group. For hypothesis 1a, there were no regions in which neural similarity significantly interacted with closeness to predict interaction success in just the non-autistic group. For the exploratory analysis assessing peer vs. non-peer differences, there were no regions in which neural similarity significantly interacted with peer interaction status to predict interaction success in just the non-autistic group.

**Exploratory Analysis: Within-Group Analyses – Autistic Group**

Next, hypotheses were tested within just the autistic group (*n*=25), using neural similarity values calculated from each autistic participant to every other autistic participant. For hypothesis 1, there were no regions across the twelve mentalizing/reward ROIs in which neural similarity significantly predicted interaction success in just the autistic group. For hypothesis 1a, there were several regions that survived correction for multiple comparisons in which neural similarity significantly interacted with closeness to predict interaction success in just the autistic group. These regions included the regions identified in the full sample, rTPJ and lOFC, as well as regions that were not identified in the full sample, specifically ACC, AMY, dmPFC, lATL, lTPJ, precuneus, vmPFC, and VS (Supplemental Table S26).

*Supplemental Table S26. Interactions Between Closeness and Neural Similarity in the Autistic Sample*

| ROI | *B*_closeXnSim_ | St. Error | df | *t* | *p*_raw_ | *p*_corrected_ |
| --- | --- | --- | --- | --- | --- | --- |
| ACC | 4.405 | 1.976 | 449.91 | 2.229 | 0.0263 | 0.0316 |
| AMY | 9.804 | 2.901 | 449.74 | 3.380 | 0.0008 | 0.0019 |
| dmPFC | 4.954 | 1.518 | 449.83 | 3.264 | 0.0012 | 0.0024 |
| lATL | 3.440 | 1.490 | 449.92 | 2.308 | 0.0214 | 0.0316 |
| lOFC | 4.270 | 1.725 | 449.91 | 2.475 | 0.0137 | 0.0235 |
| lTPJ | 6.586 | 1.139 | 449.53 | 5.784 | <0.0001 | <0.0001 |
| Precuneus | 1.860 | 0.829 | 449.86 | 2.243 | 0.0254 | 0.0316 |
| rATL | 1.109 | 1.623 | 449.90 | 0.683 | 0.4950 | 0.5399 |
| rTPJ | 6.482 | 1.083 | 449.57 | 5.986 | <0.0001 | <0.0001 |
| rVFC | -0.496 | 1.665 | 450.19 | -0.298 | 0.7661 | 0.7661 |
| vmPFC | 12.357 | 2.371 | 449.11 | 5.212 | <0.0001 | <0.0001 |
| VS | 13.144 | 3.584 | 450.02 | 3.668 | 0.0003 | 0.0008 |

Finally, there were no regions across the twelve mentalizing/reward ROIs in which neural similarity significantly interacted with peer interaction status to predict interaction success in just the autistic group.

**Whole-Brain Group Differences in Neural Similarity**

Linear mixed effects models with crossed random effects (Chen et al., 2017) were implemented to test the hypothesis that the autistic group had significantly reduced neural similarity compared to the non-autistic group (reduced intersubject correlation between autistic/autistic dyads compared to non-autistic/non-autistic dyads). Across both a whole-brain parcellation (Shen et al., 2013) and twelve mentalizing/reward ROIs, no regions survived correction for multiple comparisons.

*Supplemental Table S27. Descriptive Statistics for Neural Similarity by Group in a Set of Twelve Mentalizing- and Reward-Related ROIs.*

| ROI Neural Similarity  Mean (SD) | Autistic (*n*=25) | Non-Autistic (*n*=67) |
| --- | --- | --- |
| ACC | 0.034 (0.015) | 0.036 (0.017) |
| AMY | 0.034 (0.015) | 0.039 (0.015) |
| dmPFC | 0.074 (0.030) | 0.076 (0.036) |
| lATL | 0.070 (0.035) | 0.084 (0.030) |
| lOFC | 0.042 (0.024) | 0.039 (0.020) |
| lTPJ | 0.107 (0.049) | 0.126 (0.043) |
| Precuneus | 0.169 (0.052) | 0.184 (0.049) |
| rATL | 0.082 (0.033) | 0.092 (0.033) |
| rTPJ | 0.118 (0.051) | 0.133 (0.043) |
| rVFC | 0.036 (0.022) | 0.038 (0.022) |
| vmPFC | 0.042 (0.017) | 0.039 (0.019) |
| VS | 0.014 (0.008) | 0.016 (0.008) |

Supplemental Figure S1. Distributions and Correlations for Neural Similarity Variables of Interest.

**Descriptive Statistics and Distributions for EMA Variables of Interest**

*Supplemental Table S28. Descriptive Statistics for EMA Variables of Interest by Group*

|  | Autistic (25 participants) | | Non-Autistic (67 participants) | |
| --- | --- | --- | --- | --- |
| Response Type  (*n* responses) | Peer  (*n*=158) | Non-Peer  (*n*=489) | Peer  (*n*=762) | Non-Peer  (*n*=1471) |
| Interaction Success Mean (SD) | 73.2 (23.6) | 69.8 (22.5) | 79.5 (19.5) | 73.3 (21.5) |
| Interaction Closeness Mean (SD) | 67.0 (26.3) | 75.8 (26.9) | 73.2 (24.4) | 74.0 (22.2) |


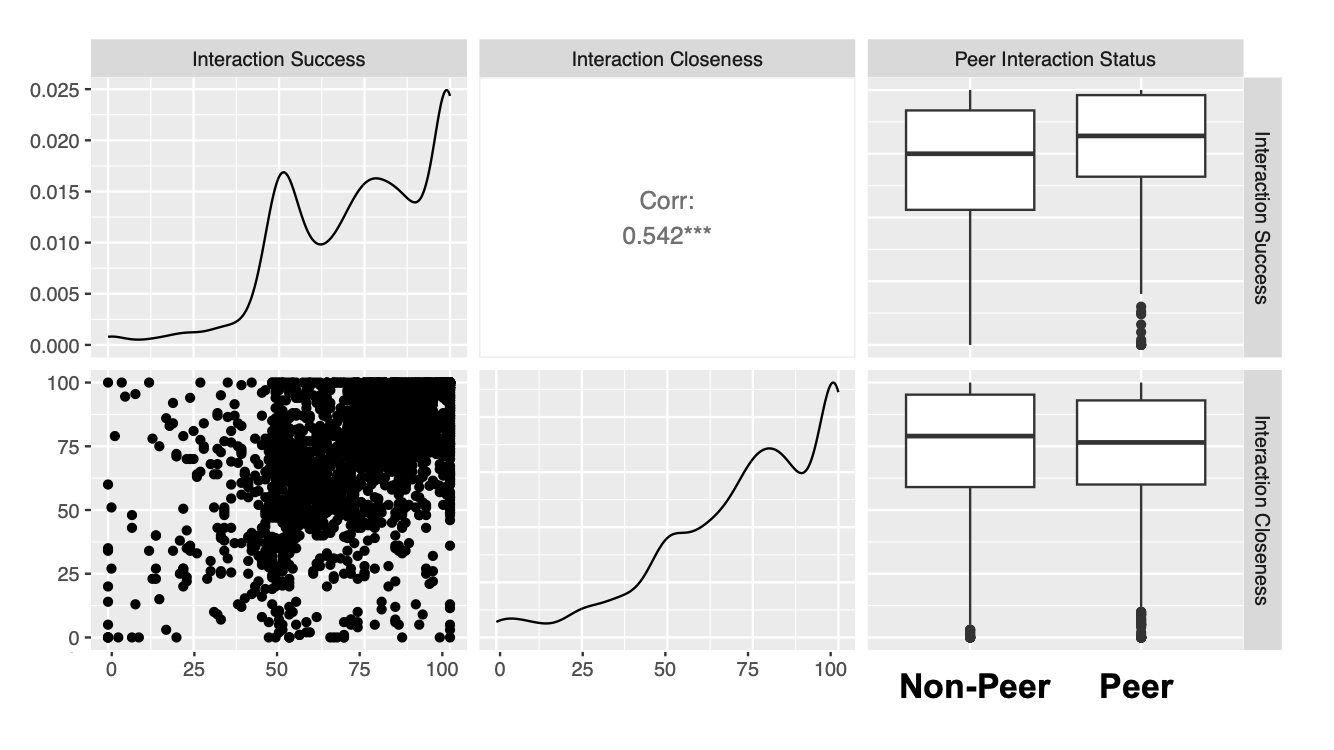
Supplemental Figure S2. Distributions and Correlations for EMA Variables of Interest.

**Supplemental Analysis: Resampling Analysis to Test Group Differences in Day-to-Day Interaction Success in Balanced AUT/NON-AUT Sample**

The autistic and non-autistic samples had unequal sample sizes, therefore, we also tested the EMA model, that is, whether the social partner (peer/non-peer) and group (autistic/non-autistic) statistically interacted to predict social interaction success in the EMA data, in a balanced sample of autistic and non-autistic participants using a resampling procedure. We resampled the participants (level 2 units) alongside all of their responses (level 1 units) to obtain a balanced autistic/non-autistic sample, in line with previous recommendations (van der Leeden et al., 2008). When this resampling procedure was run 500 times, we obtained results consistent with the results specified in the text (i.e. statistically significant interactions between group and social partner) 96% of the time.

**References**

Abraham, A., Pedregosa, F., Eickenberg, M., Gervais, P., Mueller, A., Kossaifi, J., Gramfort, A., Thirion, B., & Varoquaux, G. (2014). Machine learning for neuroimaging with scikit-learn. *Frontiers in Neuroinformatics*, *8*. https://doi.org/10.3389/fninf.2014.00014

Avants, B., Epstein, C., Grossman, M., & Gee, J. (2008). Symmetric diffeomorphic image registration with cross-correlation: Evaluating automated labeling of elderly and neurodegenerative brain. *Medical Image Analysis*, *12*(1), 26–41. https://doi.org/10.1016/j.media.2007.06.004

Behzadi, Y., Restom, K., Liau, J., & Liu, T. T. (2007). A component based noise correction method (CompCor) for BOLD and perfusion based fMRI. *NeuroImage*, *37*(1), 90–101. https://doi.org/10.1016/j.neuroimage.2007.04.042

Chen, G., Taylor, P. A., Shin, Y.-W., Reynolds, R. C., & Cox, R. W. (2017). Untangling the relatedness among correlations, Part II: Inter-subject correlation group analysis through linear mixed-effects modeling. *NeuroImage*, *147*, 825–840. https://doi.org/10.1016/j.neuroimage.2016.08.029

Cox, R. W. (1996). AFNI: Software for Analysis and Visualization of Functional Magnetic Resonance Neuroimages. *Computers and Biomedical Research*, *29*(3), 162–173. https://doi.org/10.1006/cbmr.1996.0014

Cox, R. W., & Hyde, J. S. (1997). Software tools for analysis and visualization of fMRI data. *NMR in Biomedicine*, *10*(4–5), 171–178. https://doi.org/10.1002/(SICI)1099-1492(199706/08)10:4/5<171::AID-NBM453>3.0.CO;2-L

Dale, A. M., Fischl, B., & Sereno, M. I. (1999). Cortical Surface-Based Analysis. *NeuroImage*, *9*, 179–194. https://doi.org/10.1006/nimg.1998.0395

Enders, C. K., & Tofighi, D. (2007). Centering predictor variables in cross-sectional multilevel models: A new look at an old issue. *Psychological Methods*, *12*(2), 121–138. https://doi.org/10.1037/1082-989X.12.2.121

Esteban, O., Markiewicz, C. J., Blair, R. W., Moodie, C. A., Isik, A. I., Erramuzpe, A., Kent, J. D., Goncalves, M., DuPre, E., Snyder, M., Oya, H., Ghosh, S. S., Wright, J., Durnez, J., Poldrack, R. A., & Gorgolewski, K. J. (2019). fMRIPrep: A robust preprocessing pipeline for functional MRI. *Nature Methods*, *16*(1), 111–116. https://doi.org/10.1038/s41592-018-0235-4

Esteban, O., Markiewicz, C. J., Burns, C., Goncalves, M., Jarecka, D., Ziegler, E., Berleant, S., Ellis, D. G., Pinsard, B., Madison, C., Waskom, M., Notter, M. P., Clark, D., Manhães-Savio, A., Clark, D., Jordan, K., Dayan, M., Halchenko, Y. O., Loney, F., … Ghosh, S. (2021). *nipy/nipype: 1.7.0* (Version 1.7.0) [Computer software]. Zenodo. https://doi.org/10.5281/zenodo.5585697

Esteban, O., Markiewicz, C. J., Goncalves, M., DuPre, E., Kent, J. D., Salo, T., Ciric, R., Pinsard, B., Blair, R. W., Poldrack, R. A., & Gorgolewski, K. J. (2021). *fMRIPrep: A robust preprocessing pipeline for functional MRI* (Version 20.2.6) [Computer software]. Zenodo. https://doi.org/10.5281/zenodo.5683866

Evans, A. C., Janke, A. L., Collins, D. L., & Baillet, S. (2012). Brain templates and atlases. *NeuroImage*, *62*(2), 911–922. https://doi.org/10.1016/j.neuroimage.2012.01.024

Fonov, V., Evans, A., McKinstry, R., Almli, C., & Collins, D. (2009). Unbiased nonlinear average age-appropriate brain templates from birth to adulthood. *NeuroImage*, *47*, S102. https://doi.org/10.1016/S1053-8119(09)70884-5

Gorgolewski, K., Burns, C. D., Madison, C., Clark, D., Halchenko, Y. O., Waskom, M. L., & Ghosh, S. S. (2011). Nipype: A Flexible, Lightweight and Extensible Neuroimaging Data Processing Framework in Python. *Frontiers in Neuroinformatics*, *5*. https://doi.org/10.3389/fninf.2011.00013

Greve, D. N., & Fischl, B. (2009). Accurate and robust brain image alignment using boundary-based registration. *NeuroImage*, *48*(1), 63–72. https://doi.org/10.1016/j.neuroimage.2009.06.060

Jenkinson, M., Bannister, P., Brady, M., & Smith, S. (2002). Improved Optimization for the Robust and Accurate Linear Registration and Motion Correction of Brain Images. *NeuroImage*, *17*(2), 825–841. https://doi.org/10.1006/nimg.2002.1132

Klein, A., Ghosh, S. S., Bao, F. S., Giard, J., Häme, Y., Stavsky, E., Lee, N., Rossa, B., Reuter, M., Chaibub Neto, E., & Keshavan, A. (2017). Mindboggling morphometry of human brains. *PLOS Computational Biology*, *13*(2), e1005350. https://doi.org/10.1371/journal.pcbi.1005350

Lanczos, C. (1964). Evaluation of Noisy Data. *Journal of the Society for Industrial and Applied Mathematics Series B Numerical Analysis*, *1*(1), 76–85. https://doi.org/10.1137/0701007

Power, J. D., Mitra, A., Laumann, T. O., Snyder, A. Z., Schlaggar, B. L., & Petersen, S. E. (2014). Methods to detect, characterize, and remove motion artifact in resting state fMRI. *NeuroImage*, *84*, 320–341. https://doi.org/10.1016/j.neuroimage.2013.08.048

Pruim, R. H. R., Mennes, M., Van Rooij, D., Llera, A., Buitelaar, J. K., & Beckmann, C. F. (2015). ICA-AROMA: A robust ICA-based strategy for removing motion artifacts from fMRI data. *NeuroImage*, *112*, 267–277. https://doi.org/10.1016/j.neuroimage.2015.02.064

Satterthwaite, T. D., Elliott, M. A., Gerraty, R. T., Ruparel, K., Loughead, J., Calkins, M. E., Eickhoff, S. B., Hakonarson, H., Gur, R. C., Gur, R. E., & Wolf, D. H. (2013). An improved framework for confound regression and filtering for control of motion artifact in the preprocessing of resting-state functional connectivity data. *NeuroImage*, *64*, 240–256. https://doi.org/10.1016/j.neuroimage.2012.08.052

Shen, X., Tokoglu, F., Papademetris, X., & Constable, R. T. (2013). Groupwise whole-brain parcellation from resting-state fMRI data for network node identification. *NeuroImage*, *82*, 403–415. https://doi.org/10.1016/j.neuroimage.2013.05.081

Tustison, N. J., Avants, B. B., Cook, P. A., Yuanjie Zheng, Egan, A., Yushkevich, P. A., & Gee, J. C. (2010). N4ITK: Improved N3 Bias Correction. *IEEE Transactions on Medical Imaging*, *29*(6), 1310–1320. https://doi.org/10.1109/TMI.2010.2046908

van der Leeden, R., Meijer, E., & Busing, F. M. T. A. (2008). Resampling Multilevel Models. In J. de Leeuw & E. Meijer (Eds.), *Handbook of Multilevel Analysis* (pp. 401–433). Springer New York. https://doi.org/10.1007/978-0-387-73186-5_11

Zhang, Y., Brady, M., & Smith, S. (2001). Segmentation of brain MR images through a hidden Markov random field model and the expectation-maximization algorithm. *IEEE Transactions on Medical Imaging*, *20*(1), 45–57. https://doi.org/10.1109/42.906424
